# Genome-wide and co-expression network dissection of *PgUGT-Rd1* as a central regulator of ginsenoside Rd biosynthesis in ginseng

**DOI:** 10.3389/fpls.2026.1751774

**Published:** 2026-03-02

**Authors:** Sizhang Liu, Meili Chi, Yi Wang, Meiping Zhang

**Affiliations:** 1 Jilin Ginseng Academy, Changchun University of Chinese Medicine, Changchun, China; 2 College of Life Science, Jilin Agricultural University, Changchun, Jilin, China; 3 National Ginseng Products Quality Supervision Inspection Center, Yanji, Jilin, China

**Keywords:** genome-wide association study, ginseng, ginsenoside Rd, Rd biosynthesis genes, weighted gene co-expression network analysis

## Abstract

**Introduction:**

Ginsenosides from Panax ginseng represent a major class of triterpenoid saponins with important biological activities, among which the protopanaxadiol-type ginsenoside Rd is of particular interest. Despite advances in ginsenoside research, the genetic basis and regulatory framework underlying directed Rd biosynthesis remain largely unresolved.

**Methods:**

Here, we integrated genome-wide association studies (GWAS), weighted gene co-expression network analysis (WGCNA), and multi-omics datasets across a diverse ginseng germplasm panel to identify candidate genes associated with natural variation in Rd accumulation. Expression profiling, methyl jasmonate (MeJA) induction assays, and RNA interference (RNAi)–mediated functional validation were employed to characterize the role of the key candidate gene PgUGT-Rd1 within the ginsenoside biosynthetic network.

**Results:**

Five candidate genes were identified as significantly associated with Rd content. PgUGT-Rd1 displayed strong co-expression with core enzymatic genes involved in triterpenoid saponin biosynthesis. MeJA treatment markedly induced PgUGT-Rd1 expression and enhanced Rd accumulation, whereas RNAi-mediated silencing of PgUGT-Rd1 resulted in an approximately 50% reduction in Rd levels, demonstrating its functional contribution to Rd biosynthesis.

**Discussion:**

Our findings establish PgUGT-Rd1 as an important UDP-glycosyltransferase associated with the directed biosynthesis of ginsenoside Rd and provide new insights into the regulatory architecture of ginsenoside metabolic pathways in ginseng. This integrative framework highlights candidate molecular targets for precision breeding and metabolic engineering and advances the understanding of specialized metabolism in medicinal plants.

## 1 Introduction

Ginseng (*Panax ginseng* Mey) is a major medicinal plant in traditional Chinese medicine, supported by several millennia of clinical application and a substantial body of modern pharmacological research (Wang et al., 2025). Its principal bioactive constituents, the ginsenosides, exert multiple biological effects, including immunomodulatory, anti-inflammatory, antioxidant, and antitumor activities, as well as contributing to the maintenance of nervous system homeostasis (Niu et al., 2025). On the basis of the structure of their steroidal aglycone, ginsenosides are classified into two major types: protopanaxadiol (PPD) and protopanaxatriol (PPT) (Bai et al., 2025). Among these, ginsenoside Rd, a representative PPD-type monomer, has attracted particular attention because of its potential therapeutic value in ischemia–reperfusion injury, neurodegenerative disorders, regulation of the tumor microenvironment, and abnormalities of glucose and lipid metabolism, highlighting its promising prospects for translational application (Chen et al., 2022).

In ginsenoside biosynthesis, current evidence indicates that the pathway is supplied by two isoprenoid precursor routes: the mevalonate (MVA) and the non-mevalonate (MEP) pathways. Following formation of the triterpenoid backbone, the molecules are further elaborated through a series of precisely coordinated reactions, including cytochrome P450-mediated oxidative modifications and UDP-glycosyltransferase-catalyzed glycosylation (Seki et al., 2015). Several key enzymes and regulatory nodes have been identified, such as farnesyl diphosphate synthase (PgFPS) (Hou et al., 2021), dammarenediol synthase (PgDDS) (Ni et al., 2022), P450s mediating position-specific oxidations (e.g., CYP716A53v2), and UGT family members responsible for stepwise glycosylation (e.g., UGT71A27) (Kim et al., 2018). In addition, regulatory factors controlling the biosynthesis of specific ginsenoside monomers have begun to emerge. For example, the *PgRg1–3* gene associated with Rg1 biosynthesis is induced by methyl jasmonate (MeJA), and functional evidence for its role has been obtained through RNA interference (Liu et al., 2024). Collectively, these advances provide a solid foundation for elucidating the genetic basis and molecular mechanisms underlying secondary metabolism in ginseng.

However, when focusing specifically on the biosynthesis and regulation of the individual ginsenoside Rd, current understanding remains largely extrapolated from pathway inference and structural analogy, with relatively limited evidence for specificity and systems-level organization (Ji et al., 2025). First, the rate-limiting steps and core functional genes involved in Rd biosynthesis lack systematic support from population-genetic analyses and cross-validation by integrated multi-omics datasets (Ma et al., 2024). Second, most reported candidate genes have been identified through association analyses based on total ginsenoside content or overall PPD-type abundance, making it difficult to directly translate these findings into insights on the targeted biosynthesis of Rd (Ma et al., 2024). Third, the hierarchical regulatory network linking transcription factors, structural genes, and metabolic phenotypes, the competitive or diversional relationships among intersecting pathways, and their integration with environmental cues remain poorly resolved. These knowledge gaps constrain molecular breeding and metabolic engineering strategies targeting Rd, and hinder the development of efficient and robust systems for its directed production (Jiang et al., 2024).

In recent years, the completion of whole-genome sequencing and high-quality annotation of *Panax ginseng*, together with the rapid maturation of high-throughput technologies such as diversity re-sequencing, transcriptomics, metabolomics, and phenomics, has created unprecedented opportunities to dissect complex metabolic traits (Huang et al., 2016). Genome-wide association studies (GWAS), by exploiting natural genetic variation and enabling high-resolution mapping, have achieved notable success in improving crop quality and in discovering bioactive constituents in medicinal plants (Chen et al., 2014; Du et al., 2025; Li et al., 2025; Ma et al., 2025). Integrating GWAS with weighted gene co-expression network analysis (WGCNA) allows multilayer associations among genetic variation, gene expression, and phenotypic traits to be captured at the genome-wide scale. Further combining SNP–trait associations, fine-mapping of candidate intervals, and the identification of cis- and trans-regulatory factors is expected to distill, from a complex background of signals, the key nodes most closely related to the targeted biosynthesis of Rd, thereby substantially narrowing the scope of functional validation and enhancing validation efficiency (Doonan et al., 2025).

Building on these advances and challenges, we used a diverse panel of ginseng germplasm and applied a suite of multi-omics strategies, including GWAS, WGCNA, and expression profile-based association analyses, to systematically identify candidate genes and regulatory factors significantly associated with variation in Rd content. By integrating spatiotemporal expression patterns, responses to hormone and stress treatments, and co-expression relationships, we constructed a putative regulatory network for ginsenoside Rd biosynthesis. Through functional validation at the molecular and biochemical levels, we elucidated key regulatory elements and their mechanisms of action, thereby providing new evidence for the mechanistic basis of the PPD-type ginsenoside branch pathway, offering tractable targets and markers for molecular design breeding and metabolic engineering of ginseng, and laying a theoretical and technical foundation for the high-value, sustainable production of Rd in heterologous chassis or cellular factories.

## 2 Materials and methods

### 2.1 Database

A total of five databases were used in this study: Database I: Transcriptome data from roots of 344 four-year-old cultivated accessions in the Jilin *ginseng* core germplasm collection (NCBI/GEO: SRR23758499–SRR23758802, SRR13131364–SRR13131405) (Liu et al., 2023). This dataset includes transcript sequences, gene/transcript expression levels, SNP and insertion/deletion (InDel) information, as well as ginsenoside Rd content. Database II: Genome sequence data of *Panax ginseng (*Wang et al., 2022). Database III: Root transcriptome data from the same cultivated variety at 5, 12, 18, and 25 years of age (NCBI/SRA: SRX1445580–SRX1445583), including transcript sequences and expression levels. Database (Wang et al., 2015) IV: Transcriptome data from 14 tissues of four-year-old ginseng plants at the red-fruit stage in Jilin Province, China (leaf, fruit peduncle, arm root, fruit stalk, main root cortex, stem, rhizome, leg root, leaflet petiole, fruit flesh, petiole, main root epidermis, fibrous root, and seed), providing transcript sequences and expression levels for each gene (NCBI/SRA: SRX1445566–SRX1445579) (Wang et al., 2015).Database V: Transcriptome data of ginseng adventitious roots treated with methyl jasmonate (MeJA) at different time points, including 0 h (control) and 6–120 h. This dataset provides gene transcript expression levels at each time point, together with the contents of multiple ginsenoside monomers (including Rd) (Jiang et al., 2022).

### 2.2 Identification of candidate genes involved in Rd biosynthesis

#### 2.2.1 Genome-wide association study

##### 2.2.1.1 Statistical analysis of Rd content in the core germplasm population

Rd phenotypic data were organized using Excel (Version 2016), and best linear unbiased prediction (BLUP) values were calculated in R (lme4 package). A frequency histogram of Rd content was plotted in Origin 2022, and the Kolmogorov–Smirnov (K–S) test for normality was performed in IBM SPSS Statistics.2.2.1.2 Genome-wide Association Analysis using Single-Locus Models.

##### 2.2.1.2 Single-locus model genome-wide association analysis

Based on 39,327 high-quality SNP markers obtained from root transcriptomes of 344 four-year-old accessions, single-locus association analyses were conducted in TASSEL v5.2.80 using Rd BLUP values as the phenotype (Bradbury et al., 2007). The models evaluated included GLM, GLM(Q), GLM(PCA), MLM, MLM(Q+K), and MLM(PCA+K). Manhattan plots were generated to display the genome-wide distribution of p-values, and Q–Q plots were used to evaluate model fit. Linkage disequilibrium (LD)-based preliminary filtering was performed with PLINK v1.90 (window size = 50, step size = 50, r² ≥ 0.2). The significance threshold was set by Bonferroni correction at p = 2.54 × 10^-^_7_ (−log_10_p = 6.59).

##### 2.2.1.3 Multi-locus model genome-wide association analysis

To enhance robustness, multi-locus association analyses were carried out in mrMLM v4.0.2. Significant SNPs on the same chromosome within a physical distance ≤ 20 kb were merged and considered as a single locus. Five models were employed: mrMLM (Wang et al., 2016), FASTmrMLM (Wen et al., 2018), FASTmrEMMA (Wen et al., 2020), ISIS EM-BLASSO (Tamba et al., 2017), and pLARmEB (Zhang et al., 2017). The kinship matrix was calculated in TASSEL v5.2.80, and all other parameters were kept at their default settings. The significance threshold for multi-locus detection was set at LOD ≥ 3.0.

##### 2.2.1.4 Determination of genes within significantly associated intervals in genome-wide association analysis

Only loci that were significant across all six single-locus models in 2.2.1.2, as well as loci consistently detected by all five multi-locus models in 2.2.1.3, were retained. Each such jointly significant locus was defined as a quantitative trait nucleotide (QTN). A 500-kb region upstream and downstream of each QTN was designated as the candidate interval, from which genes were extracted and defined as “Rd candidate genes I.”

#### 2.2.2 Weighted gene co-expression network analysis

A weighted gene co-expression network was constructed in R using the WGCNA package (Xu et al., 2023). The input was the expression matrix of “Rd candidate genes I”; genes with expression equal to 0 in more than 70% of accessions were removed. Sample clustering was performed with hclust to identify and remove outliers. The function pickSoftThreshold was used to select the soft-thresholding power satisfying the scale-free topology criterion (power = 8.5 in this study). The blockwiseModules function was then applied to construct the network, generate the adjacency matrix and topological overlap matrix (TOM), and identify co-expression modules. Using Cytoscape 3.9.0, the top five genes with the highest connectivity were selected from key modules as hub genes and defined as “Rd candidate genes II.”

#### 2.2.3 SNP–phenotype association between candidate gene mutations and Rd content

SNP/InDel association analysis was conducted using a filtered dataset of 4,201,232 SNP/InDel variants across 344 Panax ginseng accessions. Candidate gene II variants associated with Rd biosynthesis were extracted using VCFtools v2.1. For each SNP/InDel site within these genes, individuals were grouped by genotype. The association between SNP genotype and Rd content was evaluated using independent-samples t-tests in R, with significance defined as *P* ≤ 0.05. Variants meeting this threshold were considered significantly associated with Rd content and classified as Candidate Gene III variants.

To assess biological relevance, we calculated the phenotypic impact rate (%) of each significant variant using the formula:


Impact rate(%)=(High group mean–Low group mean)/Low group mean×100%

Additionally, functional annotations were performed using NCBI’s ORF Finder to determine whether SNPs were located in coding regions. Variants were further classified as synonymous, non-synonymous, or frameshift mutations based on their predicted effects on amino acid sequences.

All analyses were performed using default parameters in VCFtools and custom scripts in R (version 4.2). Quality control was ensured by retaining only biallelic SNPs with a minor allele frequency (MAF) ≥ 0.05 and missing data rate ≤ 10%.

#### 2.2.4 Gene co-expression network analysis

According to Zhang et al (Zhang et al., 2020)., functionally similar genes tend to form tightly interacting networks. Therefore, “Rd candidate genes III” and 15 key enzyme genes in the ginsenoside biosynthetic pathway were jointly subjected to network-based screening. Gene expression levels for these genes were extracted using Perl, followed by correlation analysis in R, and the interaction network was visualized in BioLayout Express3D. When *p* ≤ 0.05, if a candidate gene still formed a tight network with key enzyme genes, it was confirmed as an Rd candidate gene. When the threshold was further tightened to *p* ≤ 1.0 × 10^-6^, genes that remained in close network association with key enzymes were defined as “Rd key candidate genes.”

### 2.3 Functional validation of Rd key candidate genes

#### 2.3.1 MeJA-induced validation

Adventitious roots of ginseng grown on solid B5 medium were cut into small segments of approximately 3 mm and inoculated into 250 mL Erlenmeyer flasks containing 150 mL liquid B5 medium (supplemented with 0.05% IAA, 1.2 mL) at an inoculum size of 1.0 g. Cultures were maintained in the dark at 22 °C and 110 rpm. On day 25 of liquid culture, MeJA (200 μM) was added for induction. Sampling time points were 0, 6, 12, 24, 36, 48, 60, 72, 84, 96, 108, and 120 h, with three biological replicates per time point; 0 h served as the negative control. For each sampling, 2 g of adventitious roots were collected, rapidly frozen in liquid nitrogen, and stored at −80 °C for RNA extraction and transcriptome sequencing. The remaining samples were dried at 35 °C to constant weight, and dry weight was recorded for saponin extraction and content determination (corresponding to Database V).

#### 2.3.2 RNA interference validation

A reverse (antisense) fragment (100 bp) of the Rd key candidate gene was seamlessly cloned into the *BamHI/XbaI* sites of the *pFGC5941* vector, followed by insertion of the corresponding forward (sense) fragment (100 bp) into the *AscI/SwaI* sites of the same vector to construct an RNAi recombinant plasmid. The plasmid was transformed into *Agrobacterium rhizogenes* C58C1 competent cells, and positive clones were selected on YEP solid medium containing rifampicin 50 ng/µL, kanamycin 50 ng/µL, and streptomycin 50 ng/µL. The positive strains were then cultured in YEP liquid medium with the same antibiotics to OD_600_ = 0.4, centrifuged, and resuspended in 1/2 MS liquid medium containing acetosyringone (AS), and the suspension was adjusted to OD_600_ = 0.5. Pre-cultured adventitious roots were infected with the bacterial suspension for approximately 15 min. After infection, the roots were cultured in the dark for 48 h (22–25 °C) on 1/2 MS solid medium containing 20 μM AS, and then transferred to 1/2 MS medium containing cefotaxime 100 ng/µL until new roots formed. Individual root lines were then propagated and subcultured in 1/2 MS liquid medium (22 °C, 110 rpm) to establish single-root lines. Part of each sample was frozen in liquid nitrogen (−80 °C) for total RNA extraction and qRT–PCR analysis, while the remaining material was dried at 35 °C to constant weight and stored at 4 °C for saponin extraction and content determination. Gene-specific primers used for qRT–PCR analysis are listed in **Supplementary Table S6**.

#### 2.3.3 Saponin extraction and HPLC analysis

All dried root tissues from methyl jasmonate-treated samples and RNAi samples were ground into powder for ginsenoside extraction. Approximately 100 mg of dried powder per sample was extracted using 5 mL of 70% methanol solution at room temperature via ultrasonication for 30 minutes. The extract was centrifuged at 10,000×g for 10 minutes, and the supernatant was collected. This extraction step was repeated three times. The combined extracts were filtered and evaporated to dryness under reduced pressure. The residue was redissolved in 1 mL of methanol, filtered through a 0.22 μm syringe filter, and analyzed. Ginsenoside Rd content was determined by high-performance liquid chromatography (HPLC). The HPLC system comprised a Waters 2695 chromatographic system and a reverse-phase C18 column (250 mm × 4.6 mm, 5 µm particle size). The mobile phase consisted of solvent A (water) and solvent B (acetonitrile), employing a gradient elution program optimized for ginsenoside separation. Gradient conditions were as follows: 0–40 min, 18%–21% B; 40–42 min, 21%–26% B; 42–46 min, 26%–32% B; 46–66 min, 32%–33.5% B; 66–71 min, 33.5%–38% B; 71–86 min, 38%–65%B; 86–91 min, 65%B; 91–96 min, 65%–85%B; 96–103 min, 85%B; 103–105 min, 85%–18%B; 105–120 min, 18%B (column equilibration). The injection volume was 10 μL, column temperature was set at 30 °C, flow rate was 1.0 mL/min, and eluents were detected by a UV detector at 203 nm (characteristic absorption peak for dammarane-type ginsenosides). Ginsenoside Rd was qualitatively identified by comparing retention time and UV spectra with Rd standard reference material. Quantification was performed using a calibration curve established with the standard material. Each sample underwent a 120-minute HPLC run to ensure baseline separation of the Rd peak, thereby guaranteeing the accuracy, repeatability, and traceability of the analytical method.

### 2.4 Elucidation of the molecular mechanism of *PgUGT-Rd1* in Rd biosynthesis

#### 2.4.1 Functional annotation and homology analysis

*PgUGT-Rd1* was functionally annotated using Blast2GO v6.0.3, drawing on Gene Ontology (GO) and KEGG databases for putative function. GO annotation placed *PgUGT-Rd1* into three primary categories: Biological Process (BP), Cellular Component (CC), and Molecular Function (MF). At GO Level 2, *PgUGT-Rd1* was annotated under BP as participating in “organic substance metabolic process” (GO:0071704), under MF as exhibiting “glycosyltransferase activity” (GO:0016757), and under CC as an “intracellular anatomical structure” (GO:0005622). These GO terms are consistent with *PgUGT-Rd1* encoding an intracellular enzyme involved in metabolic processes (notably, glycosylation). KEGG pathway mapping associated *PgUGT-Rd1* with general metabolic pathways (e.g., glycolysis, carbon metabolism, pyruvate metabolism), although these broad assignments likely reflect basic metabolic roles rather than specific saponin biosynthesis.

To determine the identity of *PgUGT-Rd1*, we compared its sequence with known ginseng genes. In contrast to our initial exploratory comparison with *P450* genes, which showed no significant homology, we focused on UDP-glycosyltransferases (UGTs) known to function in ginsenoside biosynthesis. A BLAST alignment of the full-length *PgUGT-Rd1* amino acid sequence was performed against known ginseng UGT sequences, including *UGT71A27* and *UGT74AE2* (which are involved in glycosylation of ginsenosides at specific positions) as well as other plant UGTs. *PgUGT-Rd1* shared only ~43% amino acid identity with the closest cloned ginseng UGTs (*UGT71A27_2* and *UGT74AE2*), indicating that *PgUGT-Rd1* represents a novel UGT enzyme likely involved in Rd biosynthesis. It did not cluster into the previously characterized *UGT71* or *UGT74* families, suggesting it may belong to a different UGT subfamily. To further classify *PgUGT-Rd1*, we conducted a phylogenetic analysis: we constructed a Neighbor-Joining phylogenetic tree using the amino acid sequences of *PgUGT-Rd1* and a representative set of plant UGTs (including those from P. ginseng and other species). The phylogeny (**Supplementary Figure S6B**) confirmed that *PgUGT-Rd1* falls within the plant UGT clade but on a distinct branch, separate from *UGT71A27*, *UGT74AE2*, and *UGT94Q2*, among others. This analysis supports the conclusion that *PgUGT-Rd1* is a UDP-glycosyltransferase (UGT) superfamily member, distinct from previously characterized ginseng UGTs, and is not a cytochrome P450. We therefore designate *PgUGT-Rd1* as *PgUGT* (*Panax ginseng* UDP-glycosyltransferase) for clarity in discussions of its function.

#### 2.4.2 Expression patterns across developmental stages and varieties

Given that different transcripts of the same gene may exhibit distinct functions, expression levels of *PgUGT-Rd1* and 15 transcripts derived from 11 key enzyme genes in the ginsenoside biosynthetic pathway were extracted from Databases III, IV, and I. Heat maps were generated to evaluate the spatiotemporal expression patterns of *PgUGT-Rd1* and their similarity to those of key enzyme transcripts.

#### 2.4.3 Impact of co-expression network dynamics on Rd biosynthesis

Expression levels of *PgUGT-Rd1* and the aforementioned 15 key enzyme transcripts were extracted. Based on Rd content in 42 accessions from the core germplasm, materials were divided into three groups—low (Low), medium (Mid), and high (High) Rd content (14 accessions per group). In R 4.1.2, pairwise expression correlations among genes were calculated within each group and imported into BioLayout Express3D to construct co-expression networks. Changes in nodes and edges among networks of different groups were then compared to assess their potential impact on Rd biosynthesis.

#### 2.4.4 Analysis of the genetic effects of *PgUGT-Rd1* SNPs

In the set of 344 accessions, the *PgUGT-Rd1* SNP genotypes included TT and AA (homozygotes) and TA (heterozygote). The favorable allele was first determined as follows: if the Rd content of the TT group was higher than that of the AA group, then “T” was defined as the favorable allele and “A” as the unfavorable allele. On this basis, the genetic effect type of TA was evaluated as:TA > AA: Overdominance (OD);TA = AA: Complete dominance (CD);(AA + TT)/2 < TA < AA: Incomplete dominance (ID);TA = (AA + TT)/2: Additivity (AD);TT < TA < (AA + TT)/2: Negative incomplete dominance (NID);AA = TA: Negative complete dominance (NCD);TA< AA: Negative overdominance (NOD).

## 3 Results

### 3.1 Identification of candidate genes and key candidate genes for ginsenoside Rd biosynthesis

#### 3.1.1 Genome-wide association analysis

##### 3.1.1.1 Statistical analysis of ginsenoside Rd content

Statistical analysis of Rd content in four-year-old roots from 344 ginseng landraces in the core germplasm collection showed a range of 0.04–0.891 mg/g, with an average of 0.75 mg/g, a standard deviation of 49.1%, and a coefficient of variation of 236.2%. These results indicate marked differences in Rd content among landraces and substantial genetic variation for this trait. To reduce environmental interference and improve the accuracy of estimating this complex quantitative trait, Rd content for each accession was corrected using the BLUP (Best Linear Unbiased Prediction) method (see **Supplementary Figure S1**). The corrected data conformed to a normal distribution, suggesting that Rd content is relatively evenly distributed within the core germplasm and that the population is suitable for conducting genome-wide association studies (GWAS).

##### 3.1.1.2 Single-locus genome-wide association analysis of ginsenoside Rd content

**Figure 1:**
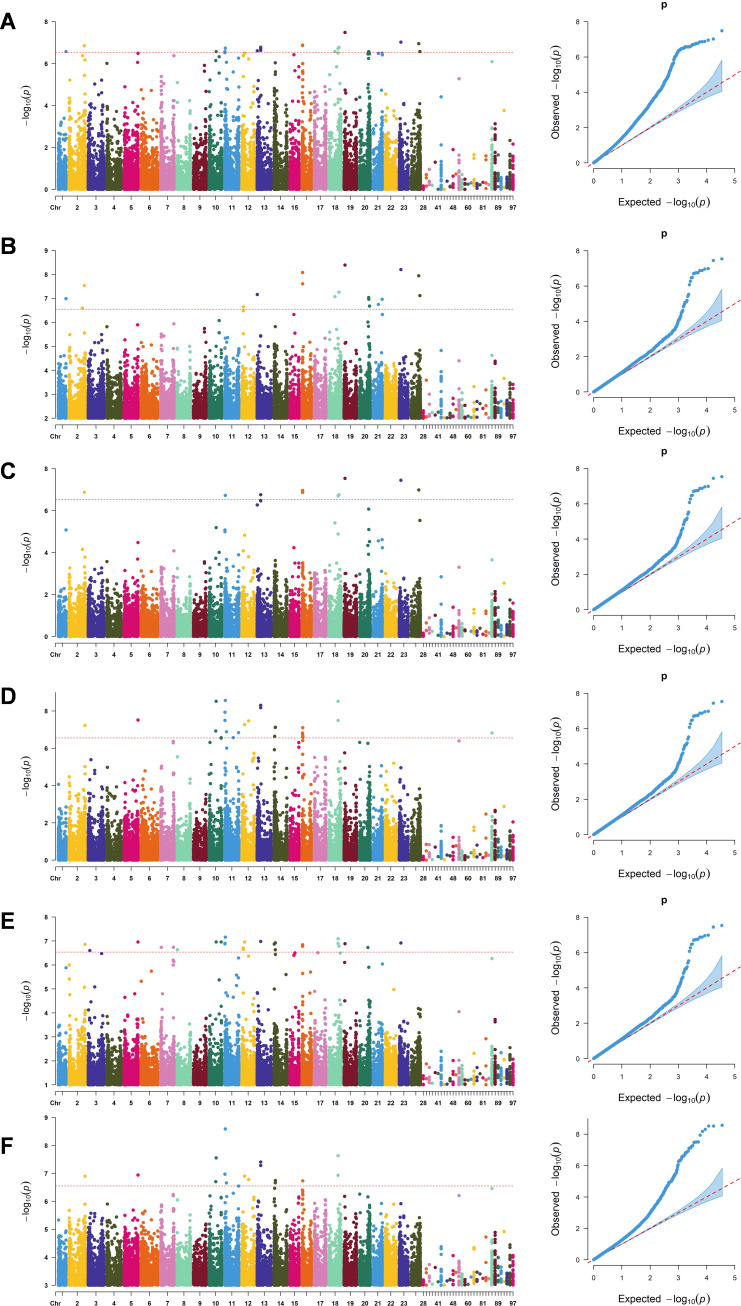
Manhattan plots of -Log10(P) vs. chromosomal position of SNP markers associated with ginsenoside Rd content and quantile-quantile (QQ) plots in a Jilin ginseng core collection using six single locus models, **(A)** GLM, **(B)** GLM(Q). **(C)** GLM(PCA). **(D)** MLM(K), **(E)** MLM(Q+K), **(F)** MLM(PCA+K). A red horizontal dashed line indicates the significant threshold -Log10(P) = 2.54E-07 from Bonferroni correction method. X-axis shows chromosomes and contigs.

Using six single-locus association models implemented in TASSEL 5 (GLM, GLM(Q), GLM(PCA), MLM(K), MLM(K+Q), and MLM(K+PCA)), genome-wide association analysis was performed with Rd content as the phenotypic trait (**Figure 1**; **Supplementary Table S1**). A total of 123 SNPs were detected that were significantly associated with Rd content, distributed across 18 chromosomes and 1 contig, each explaining 0.7–9.8% of the phenotypic variation. Among these, 67 loci were redundant, resulting in 56 unique significant association loci.

##### 3.1.1.3 Multi-locus genome-wide association analysis of ginsenoside Rd content

Using five multi-locus association models in the mrMLM 4.0 software package (mrMLM, FASTmrMLM, FASTmrEMMA, ISIS EM-BLASSO, and pKWmEB), genome-wide association analysis was conducted with Rd content as the phenotype (**Supplementary Figure S2**; **Supplementary Table S1**). In total, 53 SNPs significantly associated with Rd content were identified, distributed across 17 chromosomes and explaining 0.04–8.43% of the phenotypic variation. Among these, 24 loci were redundant, resulting in 29 unique significant association loci.

##### 3.1.1.4 Identification of genes within significantly associated genomic intervals

**Figure 2:**
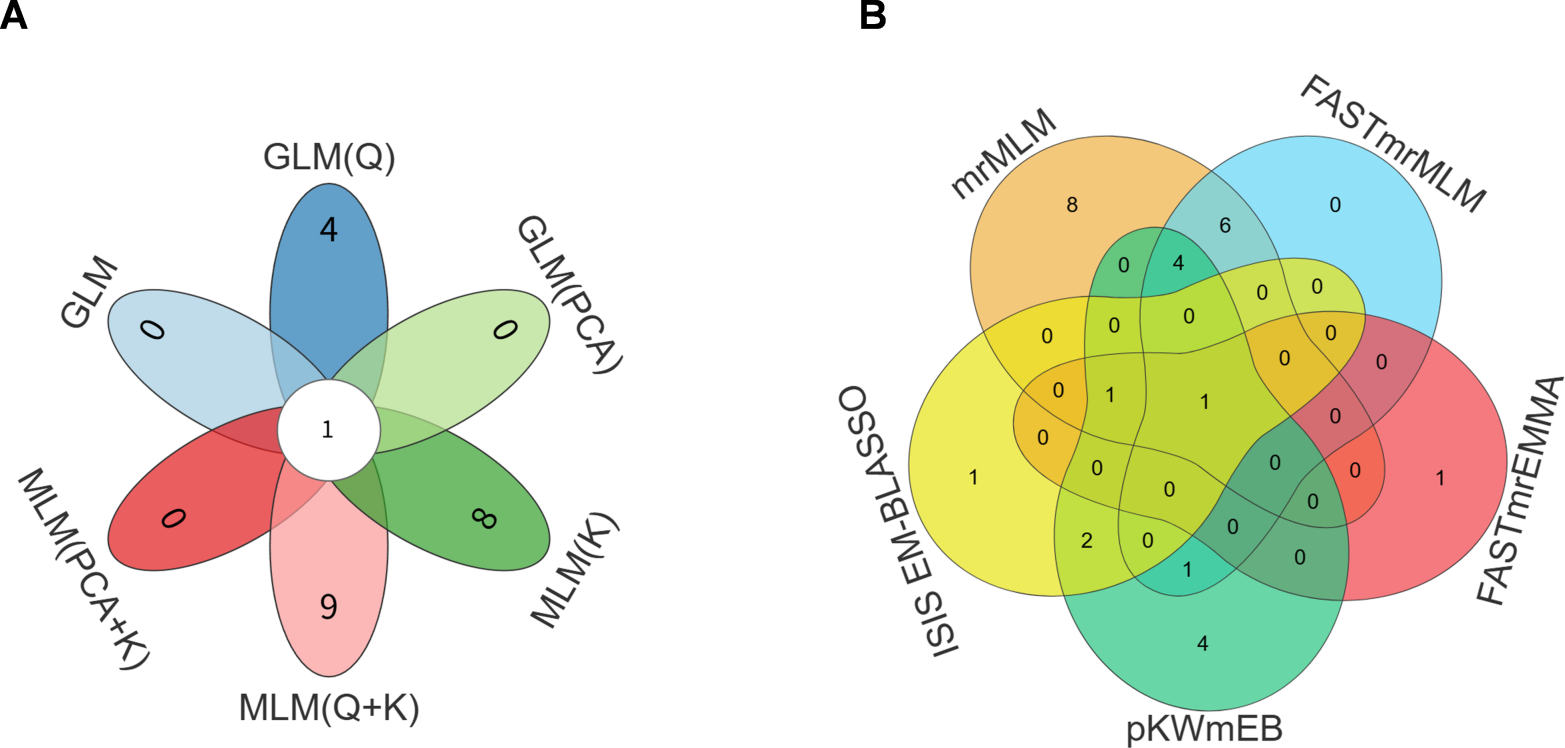
Two QTNs were consistently identified, with one by all six single locus models **(A)** and the other by all five multiple locus models **(B)**.

Combining the results of the six single-locus models and five multi-locus models, 81 loci significantly associated with Rd content were obtained. Among them, one significant association locus (QTN) was consistently detected by all six single-locus models (**Figure 2A**), and another significant association locus (QTN) was consistently detected by all five multi-locus models (**Figure 2B**). These two QTNs correspond to SNPs located at 40,077,044 bp on chromosome 13 and 4,812,374 bp on chromosome 11, respectively (**Supplementary Figure S3**). For each QTN, a 500-kb region upstream and downstream was extracted, yielding a total of 925 Rd candidate genes I (**Supplementary Table S2**).

#### 3.1.2 WGCNA analysis

Using Perl, expression data for 925 Rd candidate genes I were extracted. After removing genes with zero expression in more than 70% of samples, 909 Rd candidate genes I remained for hierarchical clustering based on expression levels. When the soft-thresholding power was set to 0.85, a total of 16 expression modules were identified (**Supplementary Figures S4A, B**). The number of genes per module ranged from 17 to 260, with the turquoise module containing the most genes (260), and the midnightblue and cyan modules containing the fewest (5 each) (**Supplementary Table S3).**

**Figure 3:**
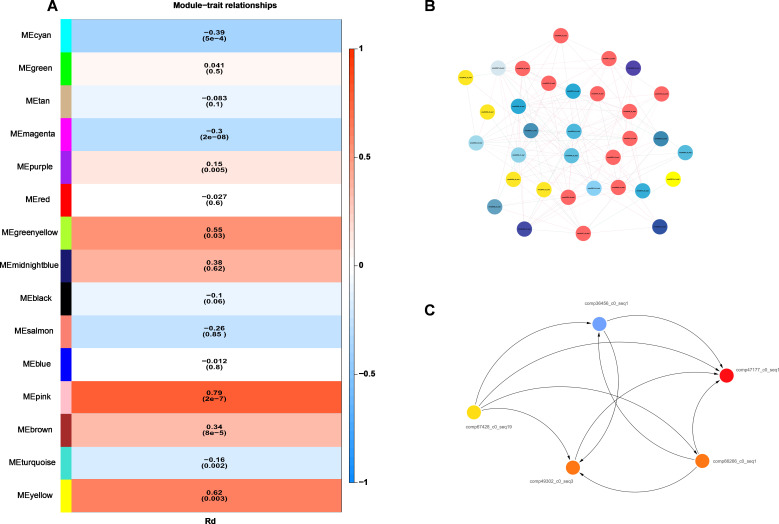
Module-trait association, orange module gene expression network and identification of hub genes. **(A)** Module-trait association. Each row corresponds to a module gene and the column to Rd content. The numbers of each row indicate correlation coefficient (") in the top and the P-value in parenthesis. **(B)** Network of Rd biosynthesis candidate genes with the highest r = 0.79 in pink module. The nodes show genes and the edges show the interactions between two genes. **(C)** Genes with top-5 degree were considered as hub genes.

Correlation analysis between these modules and Rd content showed that 7 modules were positively correlated with Rd content, while 8 modules were negatively correlated. Among them, the pink module exhibited the highest correlation coefficient (r = 0.79, *P* = 2 × 10^-^_7_; **Figure 3A**) and was designated as the key module. A weighted gene co-expression network was constructed for the 35 genes in this key module (**Figure 3B**). Genes with connectivity weight > 0.4 were retained, and the top five genes with the highest connectivity were further identified as hub genes in the pink module (**Figure 3C**). These five hub genes were defined as Rd candidate genes II.

#### 3.1.3 SNP–Rd content association analysis to assess the effect of gene mutations on Rd content

**Figure 4:**
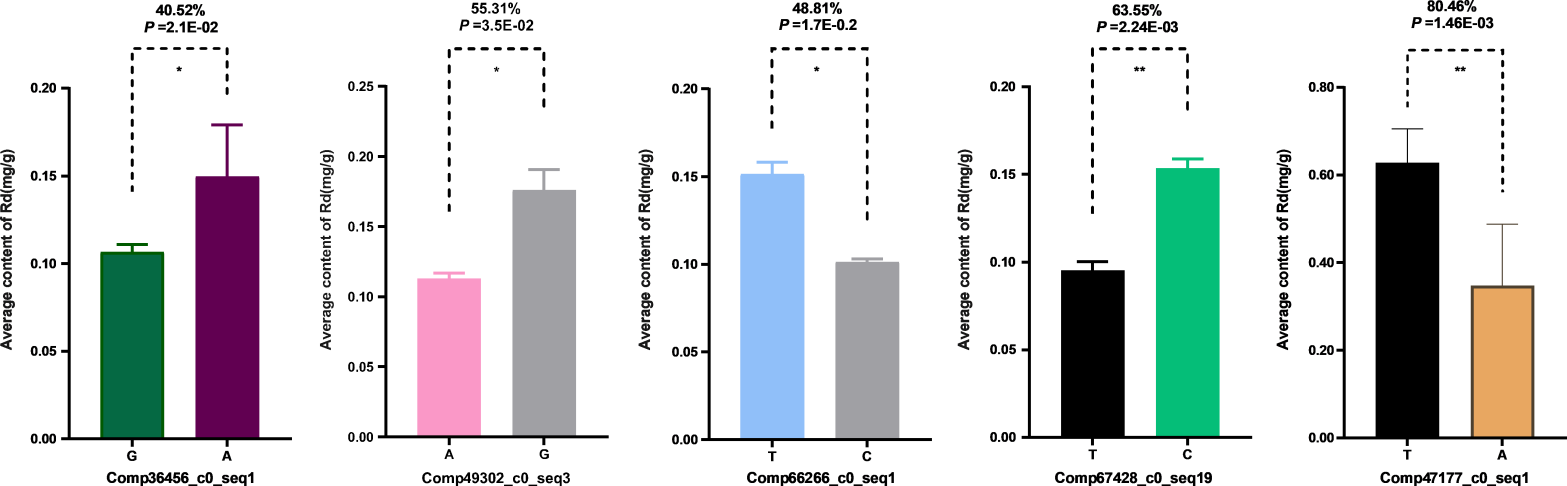
Impacts of the SNP mutations of three RD candidate genes on RD contents in roots of four-year-old plant of 344 cultivars and landraces in the ginseng core collection. The mutation of each candidate gene is shown the name and the position of the mutation. The impact of each mutation is shown percentage at the P value. For detail see Table $3. *p < 0.05; **p < 0.01.

Across the 5 Rd candidate genes II, a total of 362 SNPs were identified. Association analysis between these SNPs and Rd content showed that 37 SNPs within the 5 Rd candidate genes II were significantly associated with Rd content (*P* ≤ 0.05), accounting for 10.22% of all SNPs. Among these, 34 SNPs were located within ORFs and 3 were outside ORFs. Of the 34 SNPs within ORFs, 24 caused nonsynonymous mutations (64.86% of the total), 6 were synonymous mutations (16.22%), and 4 were ORF frameshift mutations (10.81%). The effect of each SNP on Rd content ranged from 37.57% to 80.46% (**Figure 4**; **Supplementary Table S4**). These 5 Rd candidate genes II were therefore designated as Rd candidate genes III.

#### 3.1.4 Network analysis of Rd candidate genes III and 15 key enzyme genes in ginsenoside biosynthesis

**Figure 5:**
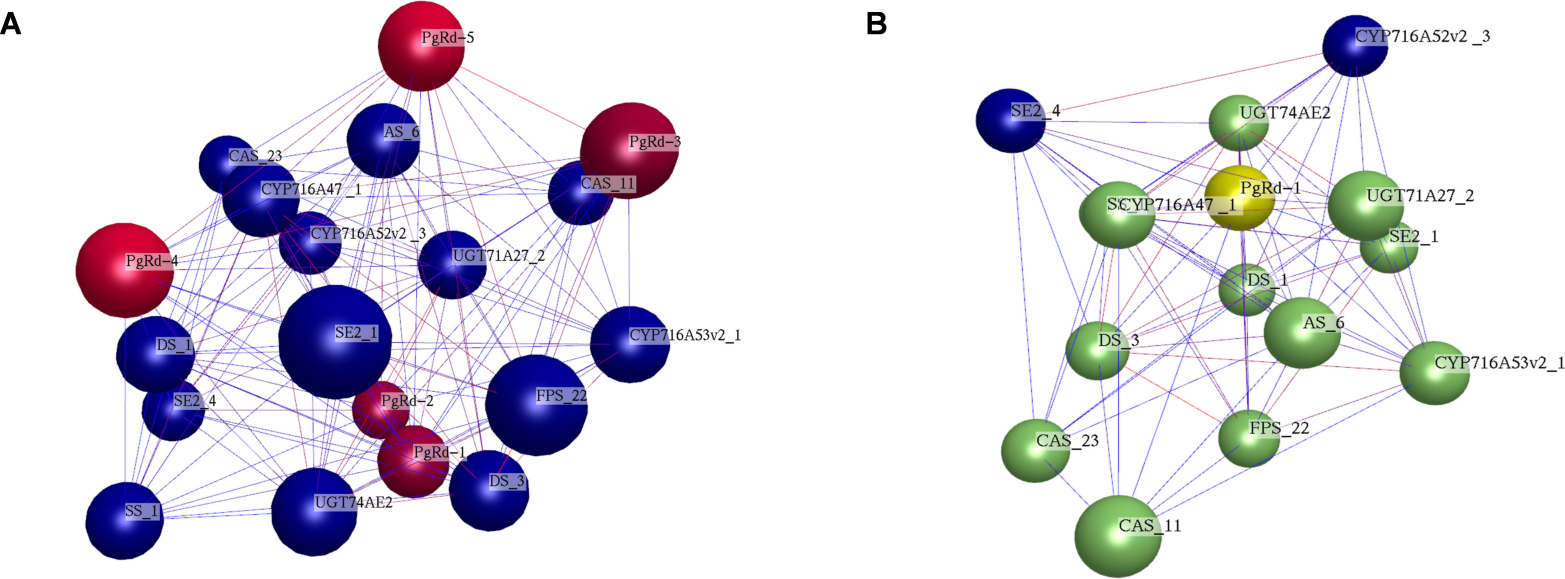
Co-expression network of three Rd candidate genes and 15 key enzyme genes involved in ginsenoside biosynthesis in four-year-old roots of 344 cultivars and landraces of the ginseng core collection. **(A)** Co- expression network of 20 genes was constructed at P < 0.05. The rhombs (nodes) represent Rd candidate genes, the balls (nodes) represent key enzyme genes for ginsenoside biosynthesis, the lines (edges) represent interactions between genes. The network consists of all five Rd candidate genes and 14 ginsenoside biosynthesis genes except B-AS_1, and two clusters showed by different colors. **(B)** Only PgRd-1 gene is in the co-expression network of 20 genes at P≤ 1.0E-08. This gene is named the Rd key candidate gene for validation of its biological function.

Using gene expression data from 344 samples, a co-expression network was constructed for the 5 Rd candidate genes III and 15 key enzyme genes involved in ginsenoside biosynthesis. The results showed that, at *P* ≤ 0.05, an interaction network comprising 20 nodes and 86 edges was formed (**Figure 5A**), indicating that these 5 Rd candidate genes III are highly likely to regulate Rd biosynthesis. These 5 Rd candidate genes III were thus confirmed as Rd candidate genes and designated *PgUGT-Rd1*, *PgRd-2*, *PgRd-3*, *PgRd-4*, and *PgRd-5*. At a more stringent *P*-value threshold of 1.0 × 10^-8^, interaction edges between the candidate gene *PgUGT-Rd1* and key enzyme genes in ginsenoside biosynthesis were still retained, suggesting a closer relationship between *PgUGT-Rd1* and ginsenoside biosynthesis in ginseng. Therefore, *PgUGT-Rd1* was selected for subsequent functional characterization (**Figure 5B**). The genomic locations, coding sequence lengths, exon–intron structures, and functional annotations of the five Rd candidate genes (PgUGT-Rd1, PgRd-2, PgRd-3, PgRd-4, and PgRd-5) are summarized in **Supplementary Table S5**.

### 3.2 Functional validation of the key candidate gene *PgUGT-Rd1*

#### 3.2.1 MeJA-induced regulation of gene expression

**Figure 6:**
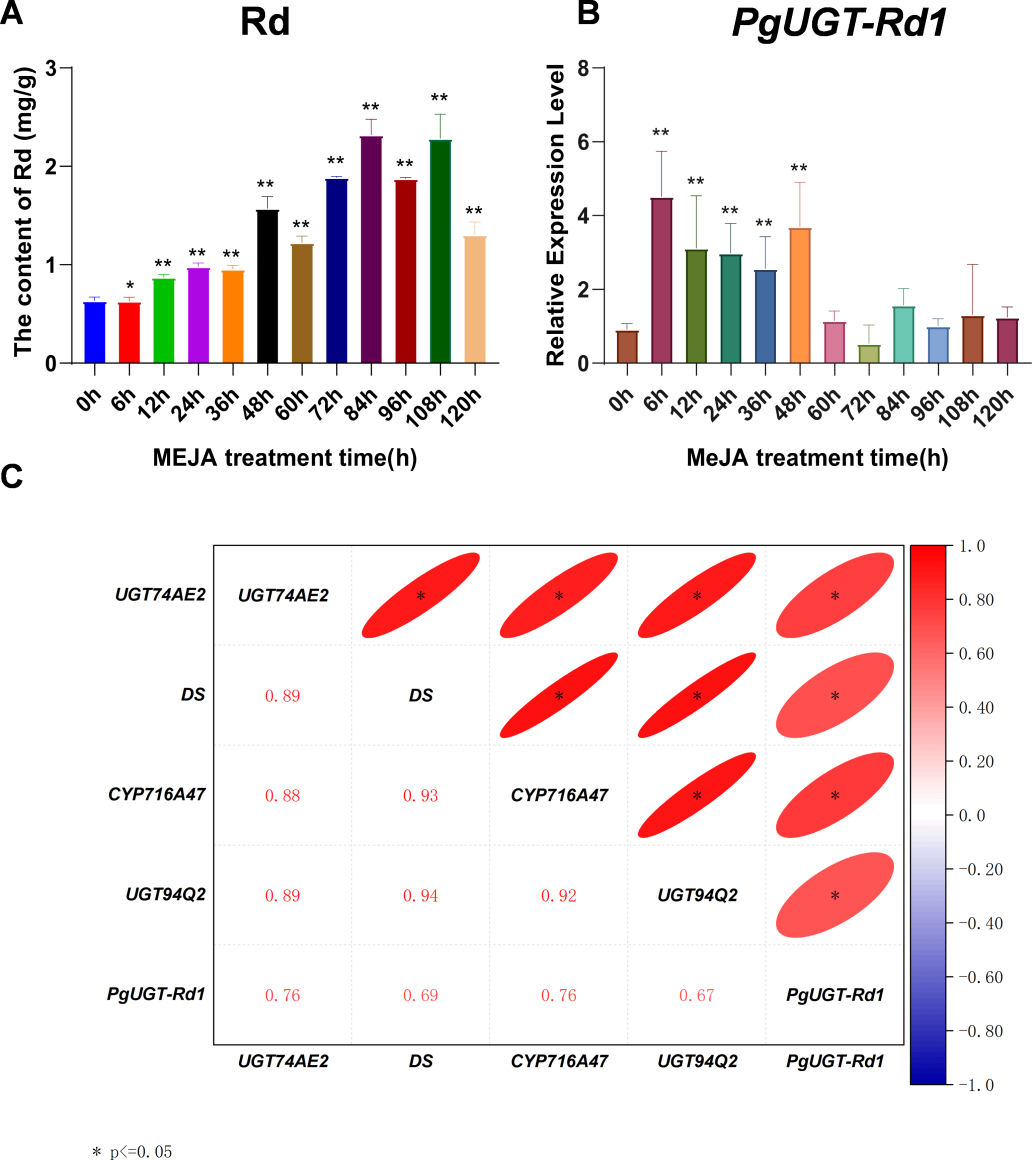
Ginseng adventitious roots treated with MeJA from 6 h to 120 h, relative to the control roots not treated with MeJA (0 h). **(A)** Rd content variations in the adventitious roots treated with MeJA, relative to the control roots. **(b)** The expression variation of PgUGT-Rdl candidate gene in the adventitious roots treated with MeJA, relative to the control roots. **(C)** Correlation of PgUGT-Rdl candidate gene and key enzyme genes involved in ginsenoside biosynthesis after MeJA inducedginseng adventitious roots. The "*" asterisks indicate the difference between MeJA treatedand control roots is significant at p < 0.05. The remaining MeJA treated rootsnot labelled with asterisk are not significantly different from the control roots. **p < 0.01.

In ginseng adventitious roots subjected to MeJA treatment for different durations, Rd content was measured by high-performance liquid chromatography. Compared with the control, Rd content showed significant changes at 6 h, 12 h, 24 h, 36 h, 48 h, 60 h, 72 h, 84 h, 96 h, 108 h, and 120 h after induction (*P* ≤ 0.05, 0.01) (**Figure 6A**), indicating that MeJA regulates Rd biosynthesis in ginseng adventitious roots.

Meanwhile, expression levels of the key candidate gene *PgUGT-Rd1* and key enzyme genes in the ginsenoside biosynthetic pathway (*CYP716A53v2_1, DS_3, ßAS_6, CAS_22, SE2_4, UGT71A27_2*) were analyzed. The results showed that *PgUGT-Rd1* expression differed significantly from the control at 6 h, 12 h, 24 h, 36 h, and 48 h after MeJA treatment (**Figure 6B**), and that the expression levels of key enzyme genes also changed significantly at certain induction time points. These findings indicate that the expression of *PgUGT-Rd1* and the key enzyme genes is not only regulated by MeJA, but that their significant expression changes also contribute to the significant changes observed in Rd content, thereby verifying the involvement of *PgUGT-Rd1* in Rd biosynthesis.

To further substantiate the role of *PgUGT-Rd1* in Rd biosynthesis, correlation analysis was conducted between *PgUGT-Rd1* and the key enzyme genes. *PgUGT-Rd1* expression was found to be significantly correlated with ßAS_6 (**Figure 6C**), providing additional evidence that *PgUGT-Rd1* participates in the biosynthesis of ginsenoside Rd.

#### 3.2.2 Genetic transformation and RNA interference of gene expression

RNAi technology was further employed to validate the function of the key candidate gene *PgUGT-Rd1*. *PgUGT-Rd1* was subjected to RNAi-mediated genetic transformation in ginseng adventitious roots (**Supplementary Figure S5**), yielding 46 positive hairy root clonal lines. Among these, five lines with stable growth were selected for determining Rd content and *PgUGT-Rd1* expression levels, using hairy roots transformed with the original *Agrobacterium rhizogenes* C58C1 strain as the negative control.

**Figure 7:**
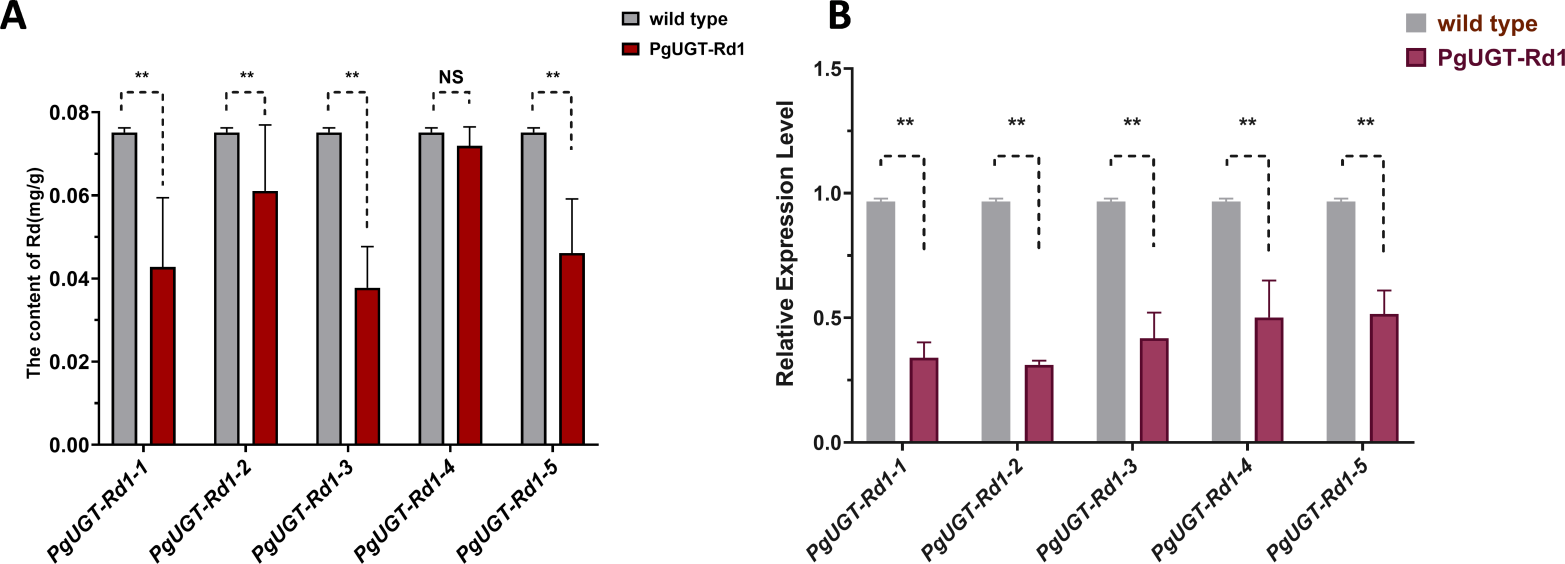
Functional validation of PgUGT-Rdl in Rd biosynthesis with RNAi genetic transformation **(A)** Rd contents in transgenic lines and WT control determined by HPLC. **(B)** Expression of PgUGT-Rdl in transgenic lines and WT control determined by qPCR. The difference in Rd content or PgUGT-Rdl expression between transgenic lines and WT control was conducted by t-test, with "**" for significanceat p < 0.01 and "NS" for non- significance.

In four of the five positive hairy root lines, Rd content was significantly reduced compared with the control, with decreases of 43.05%, 18.72%, 49.73%, and 38.62%, respectively (**Figure 7A**), indicating that *PgUGT-Rd1* regulates Rd biosynthesis. At the same time, *PgUGT-Rd1* transcript levels were significantly lower than in the control, reduced by 1.87- to 3.11-fold (**Figure 7B**), suggesting that expression of this gene was interfered with to varying degrees, thereby affecting the biosynthesis of ginsenoside Rd.

### 3.3 Exploration of the role of *PgUGT-Rd1* in Rd saponin biosynthesis

#### 3.3.1 GO functional annotation, KEGG pathway mapping, and homology comparison of *PgUGT-Rd1* with ginseng *UGT* genes

*PgUGT-Rd1* is a UDP-dependent glycosyltransferase(*UGT*) gene (*PgUGT*), with a cDNA full length of 1,390 bp and a genomic length of 2,356 bp, containing 4 exons and 2 introns. GO functional annotation of *PgUGT-Rd1* placed it into three categories: Biological Process (BP), Cellular Component (CC), and Molecular Function (MF). At Level 2, it was annotated as being involved in membrane components (organic substance metabolic process, GO:0071704), monooxygenase activity (glycosyltransferase activity, GO:0016757), and oxidoreductase activity (intracellular anatomical structure, GO:0005622). KEGG metabolic pathway annotation mapped *PgUGT-Rd1* to glycolysis, carbon metabolism, and pyruvate metabolism (**Supplementary Figure S6A**).

Phylogenetic tree of UGT family members in *Panax ginseng* based on full-length amino acid sequences. The tree was constructed using protein sequences of *PgUGT-Rd1* and several known UDP-glycosyltransferases, including *UGT71A27_2* and *UGT74AE2*, along with other PgUGT members. *PgUGT-Rd1* clusters closely with *UGT71A27_2* and *UGT74AE2*, suggesting it belongs to the same subfamily within the UGT superfamily and may share similar glycosylation functions. Phylogenetic analysis was performed using the Neighbor-Joining method, with a tree scale of 0.1. (**Supplementary Figure S6B**).

#### 3.3.2 Spatiotemporal expression patterns and activity in different landraces

In ginseng roots of different ages (5, 12, 18, and 25 years), the expression profiles of *PgUGT-Rd1* and 15 key enzyme genes involved in ginsenoside biosynthesis are shown in **Supplementary Figure S7**. The expression level of *PgUGT-Rd1* in roots of different ages followed the order: 25 years > 12 years > 5 years > 18 years. Among them, *PgUGT-Rd1* expression in 25-year-old roots was 1.22-fold that of 18-year-old roots. Overall, *PgUGT-Rd1* expression did not show a continuously increasing trend with plant age.

Similarly, the 15 key enzyme genes involved in ginsenoside biosynthesis did not exhibit a consistent age-related pattern in their expression levels across roots of different ages. Further analysis revealed that *PgUGT-Rd1* shared partially similar expression patterns with several key enzyme genes in samples of different ages: in 5-year-old roots, its expression was similar to that of *CAS_11* and *DS_1*; in 12-year-old roots, to *DS_1* and *CYP716A53v2_1*; in 18-year-old roots, to *DS_1* and *SE2_1*; and in 25-year-old roots, to *DS_1, SS_1*, and *SE2_4*. Cluster analysis grouped *PgUGT-Rd1* together with DS_1, suggesting that they may perform similar biological functions in the ginsenoside biosynthetic pathway.

Further analysis indicated that key enzyme genes with expression patterns similar to *PgUGT-Rd1* (*CAS, DS, SE, CYP716A53v2*) all occupy critical positions in aglycone formation within the ginsenoside biosynthetic pathway (**Supplementary Figure S7A**), implying that *PgUGT-Rd1* may likewise participate in aglycone biosynthesis and play an important regulatory role.

In 14 tissue types of 4-year-old ginseng roots, the expression patterns of *PgUGT-Rd1* and the 15 key enzyme genes are shown in **Supplementary Figure S7**. The expression activity of *PgUGT-Rd1* in different tissues followed the order: fibrous root = rhizome neck = stem = major petiole = minor petiole = fruit peduncle = fruit stalk = fruit flesh = seed < lateral root < leaf blade < rhizome head < root core < root periderm. Its expression was significantly higher in underground tissues than in aerial parts, with the highest activity observed in the root periderm.

The 15 key enzyme genes in ginsenoside biosynthesis could be broadly divided into two groups based on their tissue-specific expression: one group, represented by *FPS_22*, showed higher expression in underground tissues; the other, represented by *CAS_11*, was more strongly expressed in aerial parts (**Supplementary Figure S7B**). The expression pattern of *PgUGT-Rd1* was largely consistent with that of most key enzyme genes (except *CAS* and *β-AS_1*), showing higher expression in underground tissues, which suggests that Rd-type ginsenosides are likely synthesized mainly in the roots. In addition, *PgUGT-Rd1* clustered closely with *SE2_1*, indicating that the two genes may act in concert to regulate Rd biosynthesis.

In 4-year-old roots of 42 ginseng landraces, the expression patterns of *PgUGT-Rd1* and the 15 key enzyme genes are shown in **Supplementary Figure S7C**. *PgUGT-Rd1* expression varied considerably among landraces and did not display a uniform trend; notably, its expression was relatively high in varieties S2, S31, and S41. By contrast, the 15 key enzyme genes showed smaller fluctuations in expression among varieties, although in variety S23 most genes exhibited relatively high expression activity.

#### 3.3.3 Effects of changes in the gene co-expression interaction network on Rd biosynthesis

Based on the ginsenoside Rd content in roots of 42 ginseng landraces, the accessions were divided into three groups: a high-Rd-content group (High, 14 landraces), a medium-Rd-content group (Mid, 14 landraces), and a low-Rd-content group (Low, 14 landraces). Co-expression interaction network analyses were then performed separately for *PgUGT-Rd1* and 15 known key enzyme genes involved in ginsenoside biosynthesis in each group.

The results showed that the gene interaction network in the High group was the most complex, with a markedly greater number of edges than in the Mid and Low groups. Compared with the Low group, the High group had 4 more nodes and 50 more edges in its network, indicating denser and more complex interaction relationships. At the same time, the interaction degree (number of connections) between *PgUGT-Rd1* and other key enzyme genes decreased markedly from 9 in the High group to 3 in the Low group, suggesting that the tightness of interactions weakened substantially as Rd content decreased.

From the perspective of chemical composition, there were significant differences in Rd content among the three groups. The average Rd content was 0.735 mg/g in the High group, 0.549 mg/g in the Mid group, and 0.337 mg/g in the Low group. Compared with the High group, Rd content in the Low group decreased by approximately 218.13%.

**Figure 8:**
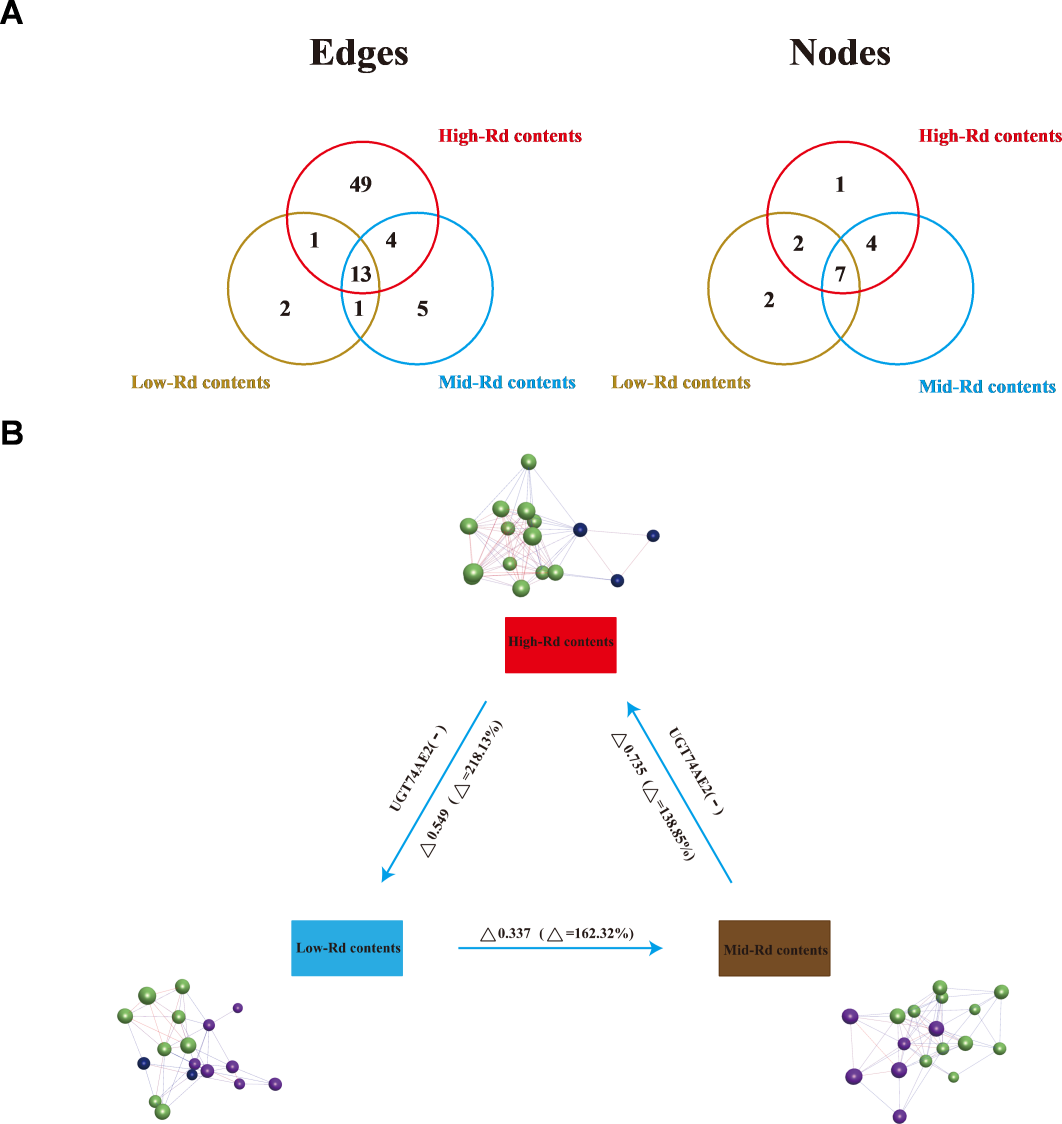
Effect of co-expression network variation of PgUGT-Rdl and 15 key enzyme genes for ginsenoside biosynthesis on Rd content among cultivar groups with high-, mid-, and low-Rd content. **(A)** Effect of variation of the gene nodes and the gene interaction edges in the network on Rd content. High-Rd content group was shown by red color, mid-Rd content group by brown color, and low-Rd content group by blue color. **(B)** Effect of number and type of genes and edges among the networks with high- , mid-, and low-Rd content groups.

Taken together, these findings indicate that the interaction network between *PgUGT-Rd1* and key enzyme genes in ginsenoside biosynthesis not only participates in regulating Rd biosynthesis, but that the complexity of this interaction network is positively correlated with Rd content. This result further supports the hypothesis that *PgUGT-Rd1* plays an important regulatory role in the Rd biosynthetic pathway (**Figure 8**).

#### 3.3.4 Effects of *PgUGT-Rd1* SNP mutations on Rd ginsenoside biosynthesis and their genetic behavior

To further elucidate the molecular mechanism by which *PgUGT-Rd1* participates in Rd saponin biosynthesis, we systematically analyzed the impact of single-nucleotide polymorphism (SNP) mutations in *PgUGT-Rd1* on Rd accumulation and their genetic effects. At position 148 bp of the *PgUGT-Rd1* gene sequence, a T→A substitution was detected, resulting in an amino acid change from glutamic acid (Glu) to histidine (His), which constitutes a nonsynonymous mutation. This mutation significantly affected Rd content: the average Rd content associated with the T allele was 0.512 mg/g, whereas that associated with the A allele was 0.348 mg/g, representing a decrease of 47.12%. Accordingly, T was defined as the favorable allele and A as the unfavorable allele. In the heterozygous genotype (TA), Rd content was 22.64% higher than in the homozygous favorable genotype (TT), indicating that this locus exhibits a completely dominant genetic effect.

In addition, a T→C substitution was identified at 400 bp of *PgUGT-Rd1*, causing an amino acid change from aspartic acid (Asp) to lysine (Lys), which is likewise a nonsynonymous mutation. This mutation led to a marked increase in Rd content: the Rd level associated with the C allele was 42.99% higher than that associated with the T allele. Thus, C was classified as the favorable allele and T as the unfavorable allele. In landraces with the heterozygous genotype (TC), Rd content was 15.7% higher than in those with the TT genotype, but 23.59% lower than in those with the CC genotype, indicating that this locus displays an incompletely dominant genetic effect with a negative dominance component. These results are summarized in **Supplementary Table S4**.

**Figure 9:**
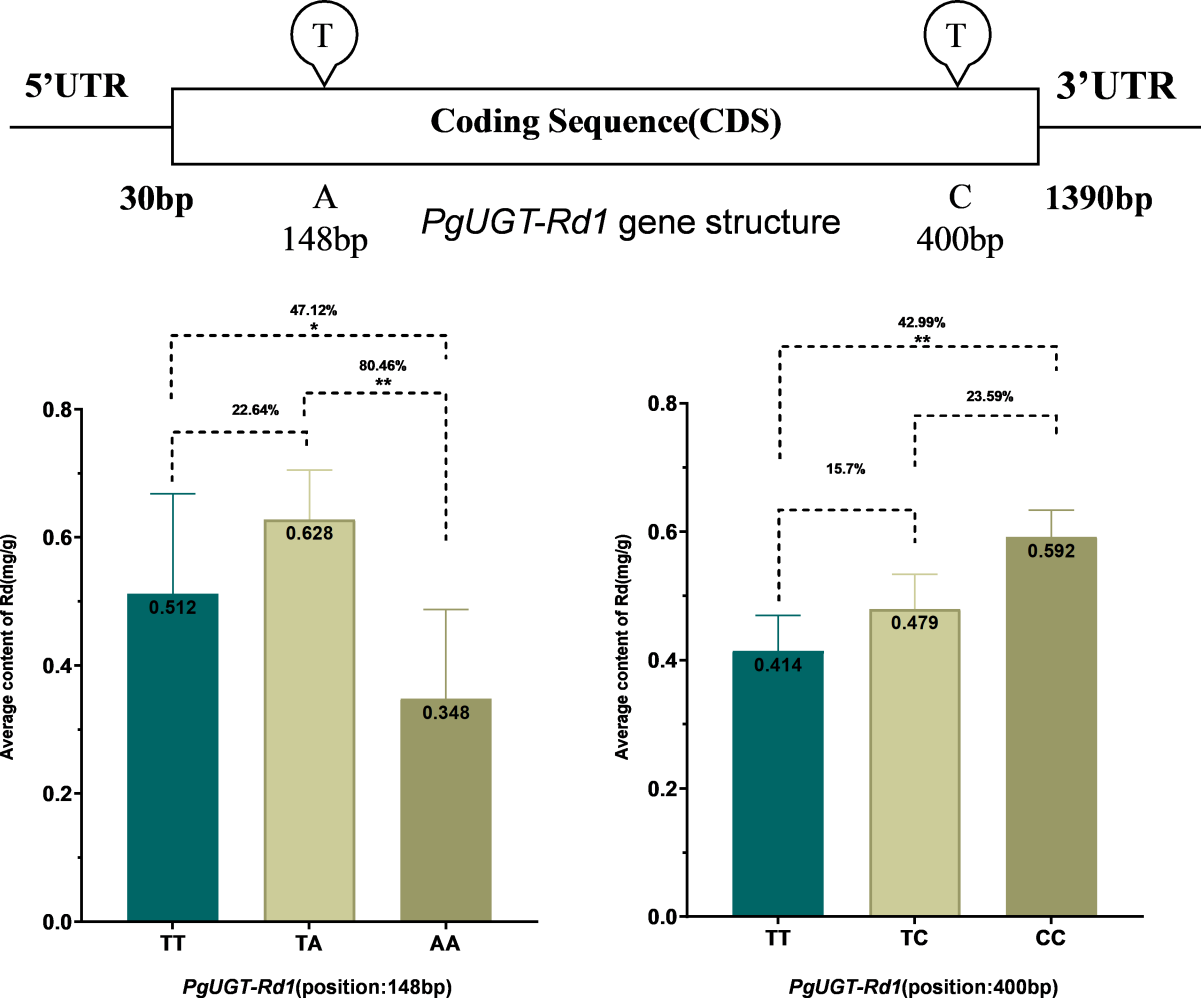
The structure of PgUGT-Rd1 gene and the negative over-dominant effect of its SNP mutation at position 148 bp and 400bp on Rd content in the 344 cultivars and landraces of Jilin ginseng core collection. The genotypes of SNP mutation are shown on the x-axis. The number in the bar presents Rd content mean in a genotype and percentage shows impact of SNP mutation on Rd content. "*" for significance level of P < 0.05, ***" for significance level of P < 0.01.

In summary, nonsynonymous SNP mutations at different sites in *PgUGT-Rd1* not only significantly influence the efficiency of Rd saponin biosynthesis, but their distinct genetic behaviors (complete dominance and incomplete dominance) further underscore the important regulatory role of *PgUGT-Rd1* in Rd biosynthesis and highlight its potential value as a target for molecular breeding (see **Figure 9**).

## 4 Discussion

This study employed an integrative, multi-omics strategy—combining GWAS, WGCNA, targeted co-expression network analysis, and transgenic functional validation—to systematically dissect the genetic regulatory mechanisms underlying ginsenoside Rd biosynthesis in *Panax ginseng*. This approach provided both methodological power and conceptual depth (Xu et al., 2025; Zhang et al., 2025). First, GWAS leveraged natural genetic variation to finely map quantitative trait loci associated with Rd-related phenotypes, establishing a solid population-genetic basis for resolving a complex secondary metabolic trait. Second, incorporating WGCNA explicitly coupled gene expression patterns with phenotypic variation, facilitating the identification of co-expression modules and core candidate genes closely associated with Rd content at the genome-wide scale (Wang et al., 2025; Wu et al., 2025). By integrating these methods, we avoided the uncertainty of inferring Rd biosynthesis genes from total ginsenoside or total PPD-type ginsenoside data, instead targeting the specific monomer Rd. This focused strategy substantially narrowed the scope of functional assays and improved the efficiency and accuracy of candidate gene discovery compared to traditional approaches based on pathway inference or structural analogy. Notably, similar frameworks have been successfully applied in other species (e.g., soybean) for mapping and validating major-effect metabolic genes, underscoring the broad utility of our approach (Azam et al., 2023; Yang et al., 2024). Here, we introduced this integrated strategy to ginseng for the first time, markedly enhancing the resolution at which the regulatory network underlying Rd biosynthesis could be elucidated.

Our findings also provided supportive evidence consistent with prior reports on ginsenoside biosynthesis, lending confidence to the results. For example, the CYP716A53v2 gene, known to catalyze the hydroxylation of protopanaxadiol to form protopanaxatriol, diverts intermediates into the PPT-type branch of the pathway (Han et al., 2012). Likewise, UGT71A27, which catalyzes glycosylation at the C-20 position of ginsenosides, contributes to the structural diversification of both PPD- and PPT-type ginsenosides (Han et al., 2011; Yan et al., 2014). Neither of these enzymes directly performs the final steps of Rd biosynthesis, yet our GWAS and co-expression analyses identified them as significantly correlated with Rd accumulation. This may reflect broader co-regulatory mechanisms within the ginsenoside metabolic network or shared transcriptional responses to stimuli such as MeJA induction. The inclusion of such genes from related pathway branches underscores the reliability of our multi-omics strategy for pinpointing relevant biosynthetic genes, even those in distinct but interconnected branches of the pathway (Han et al., 2013; Jung et al., 2014). These observations reinforce that our approach successfully distilled true positive candidates from a complex background of signals.

Most importantly, this study was the first to clearly define the role of a previously unresolved candidate gene, *PgUGT-Rd1*, as a central regulator in the Rd biosynthetic pathway. Earlier reports noted that this gene is upregulated by methyl jasmonate (MeJA) and that its RNAi-mediated suppression affects total ginsenoside levels, but its molecular identity and specific function had remained unclear (Han et al., 2006). Through multi-omics positional analysis and a series of functional validations, we identified *PgUGT-Rd1* as encoding a UDP-dependent glycosyltransferase likely involved in the dedicated glycosylation step of Rd biosynthesis, and we formally designated this locus *PgUGT-Rd1*. The expression level of *PgUGT-Rd1* was found to be highly positively correlated with Rd accumulation: MeJA treatment markedly induced *PgUGT-Rd1* expression, accompanied by a pronounced increase in Rd content, whereas RNAi-mediated silencing of *PgUGT-Rd1* reduced Rd content by approximately 50%. Taken together, these results provide the first systematic demonstration of the central role of *PgUGT-Rd1* in the specific biosynthesis of ginsenoside Rd, thereby filling a key knowledge gap in ginsenoside biology (Jiang et al., 2024).

Additional evidence from co-expression network comparisons strongly reinforced the central regulatory influence of *PgUGT-Rd1*. When we constructed gene interaction networks separately for high-, medium-, and low-Rd producing landraces, we observed that the *PgUGT-Rd1*-centered network in high-Rd genotypes was markedly more complex and connected than in low-Rd genotypes. Specifically, the high-Rd group’s network contained more nodes and ~50 additional edges compared to the low-Rd group, and the number of direct connections between *PgUGT-Rd1* and other key enzyme genes dropped from 9 in high producers to only 3 in low producers. In other words, *PgUGT-Rd1* was a highly connected hub in the genetic background of high-Rd landraces, whereas its network interactions were substantially sparser in low-Rd backgrounds. This positive correlation between network complexity and Rd content (with network density essentially tracking the level of Rd accumulation across groups) suggests that robust co-expression interactions among Rd-pathway genes are a hallmark of high-Rd producers. There are several possible explanations for these differential co-expression structures. Genetically, high-Rd landraces may carry other complementary alleles or regulatory loci that enhance the coordinated expression of *PgUGT-Rd1* and its partner genes, whereas low producers might lack such enhancers or carry variants that disrupt network integrity. Differences in cis-regulatory elements could underlie the stronger transcriptional co-regulation in high producers, leading to a tighter coupling of *PgUGT-Rd1* with upstream and downstream pathway genes. Epigenetic factors might also play a role; for instance, chromatin states permissive of high expression in certain genotypes could result in a more synchronized activation of the network. Additionally, environmental or physiological modulation could contribute to these differences – high-Rd varieties might be more responsive to endogenous signals or external cues (such as MeJA or other stresses), thereby activating *PgUGT-Rd1* and its co-expressed genes in concert, whereas low-Rd varieties show a dampened response. This finding, that network “tightness” parallels metabolic output, further supports *PgUGT-Rd1* as a crucial regulatory node: a strongly interactive *PgUGT-Rd1* is associated with efficient Rd biosynthesis, while weakened *PgUGT-Rd1* interactions coincide with poor Rd accumulation (**Figure 8**). It will be interesting for future studies to dissect these network differences in more detail, to determine which factors (genetic or environmental) are causative in shaping the co-expression network topology around *PgUGT-Rd1*.

The discovery of *PgUGT-Rd1* also provides a practically valuable molecular target for genetic improvement and breeding of high-Rd ginseng varieties. Allelic variation at this locus has a significant impact on Rd biosynthesis and shows clear potential as a selection marker. In our diversity panel, we identified distinct nonsynonymous single-nucleotide polymorphisms (SNPs) in *PgUGT-Rd1* that were associated with substantial differences in Rd content (**Figure 9**; **Supplementary Table S4**). For example, at position 148 bp of the *PgUGT-Rd1* coding sequence, a T→A mutation results in an amino acid change from glutamic acid to histidine (E→H). Genotypes carrying the A allele at this position exhibited markedly lower Rd accumulation than those with the T allele. On average, the unfavorable A allele was associated with a ~47% decrease in Rd content relative to the favorable T allele, making T the favorable allele at this locus. Notably, this site displayed a completely dominant genetic effect: heterozygotes (T/A) showed ~22% higher Rd content than even the T/T homozygotes, indicating an overdominance or heterozygote advantage for the 148 bp locus. In contrast, a T→C substitution at 400 bp of *PgUGT-Rd1* (Aspartic acid to Lysine, D→K) had the opposite effect on Rd levels. Here, the C allele was favorable, conferring an ~43% higher Rd content compared to the T allele. For this 400 bp SNP, *incomplete dominance* was observed: heterozygous T/C individuals had intermediate Rd levels (about 15–16% higher than T/T but ~23% lower than C/C), suggesting a dosage effect of the favorable allele with a slight negative dominance component. These genetic effects indicate that different allelic combinations at *PgUGT-Rd1* lead to substantial differences in Rd yield. From a biochemical perspective, the amino acid substitutions (E148H and D400K) likely influence the enzyme’s activity, stability, or interaction with substrates, thereby altering the efficiency of Rd biosynthesis. A higher-activity variant (e.g., the 400C allele) may glycosylate the precursor more efficiently or at a higher rate, boosting Rd production and perhaps strengthening the metabolic flux through the pathway, whereas a less efficient variant (e.g., 148A allele) could bottleneck the pathway. It is also plausible that *PgUGT-Rd1* allelic variants differ in expression levels or enzyme stability in planta, which could contribute to the observed differences in co-expression network connectivity (with favorable alleles supporting stronger co-expression with other pathway genes). In practical terms, the favorable alleles at *PgUGT-Rd1* (such as 148T and 400C) represent valuable molecular markers for breeding: they can be used to screen germplasm and select parental lines predicted to produce higher Rd. The fact that the 148 bp locus exhibits overdominance also presents an opportunity for hybrid breeding strategies, wherein crossing two different allelic types could produce heterozygous offspring with superior Rd content. In summary, *PgUGT-Rd1* genetic variation can be exploited to enable early selection and precise pyramiding of desirable alleles, accelerating the development of elite, high-Rd ginseng varieties.

Beyond its value in breeding, *PgUGT-Rd1* and its associated network illuminate the broader regulatory architecture of Rd biosynthesis, offering new avenues for metabolic engineering. As a core regulatory factor, *PgUGT-Rd1* functions within a three-tier framework of “transcription factor – structural gene – metabolic product” that we propose for the Rd pathway. In our co-expression network, *PgUGT-Rd1* clustered with multiple known enzyme-encoding genes of the ginsenoside pathway (such as *CAS*, *DS*, and *β-AS*) as well as candidate transcription factors, suggesting it sits at a nexus linking upstream regulatory signals to downstream metabolic outputs. Notably, *PgUGT-Rd1* showed strong co-expression with those upstream structural genes, and may act as a nodal regulator within the protopanaxadiol-type ginsenoside branch pathway (Han et al., 2013). This means that manipulating *PgUGT-Rd1* could have cascading effects on the entire module of Rd biosynthesis genes. From an applied perspective, our constructed network provides a blueprint for designing heterologous expression systems (e.g., yeast or plant cell factories) to achieve efficient Rd production. In addition to introducing the essential structural enzyme genes of the pathway into such systems, incorporating regulatory factors like *PgUGT-Rd1* could be key to optimizing metabolic flux and dynamically balancing intermediate and product levels. For instance, a yeast chassis engineered to produce Rd might achieve much higher yields if *PgUGT-Rd1* is co-expressed to enhance flux through the final glycosylation step and to coordinate with upstream steps, rather than expressing only the enzymes for each step. Thus, the regulatory network map emerging from this study lays an important foundation for synthetic biology and metabolic engineering efforts aimed at Rd production.

**Figure 10:**
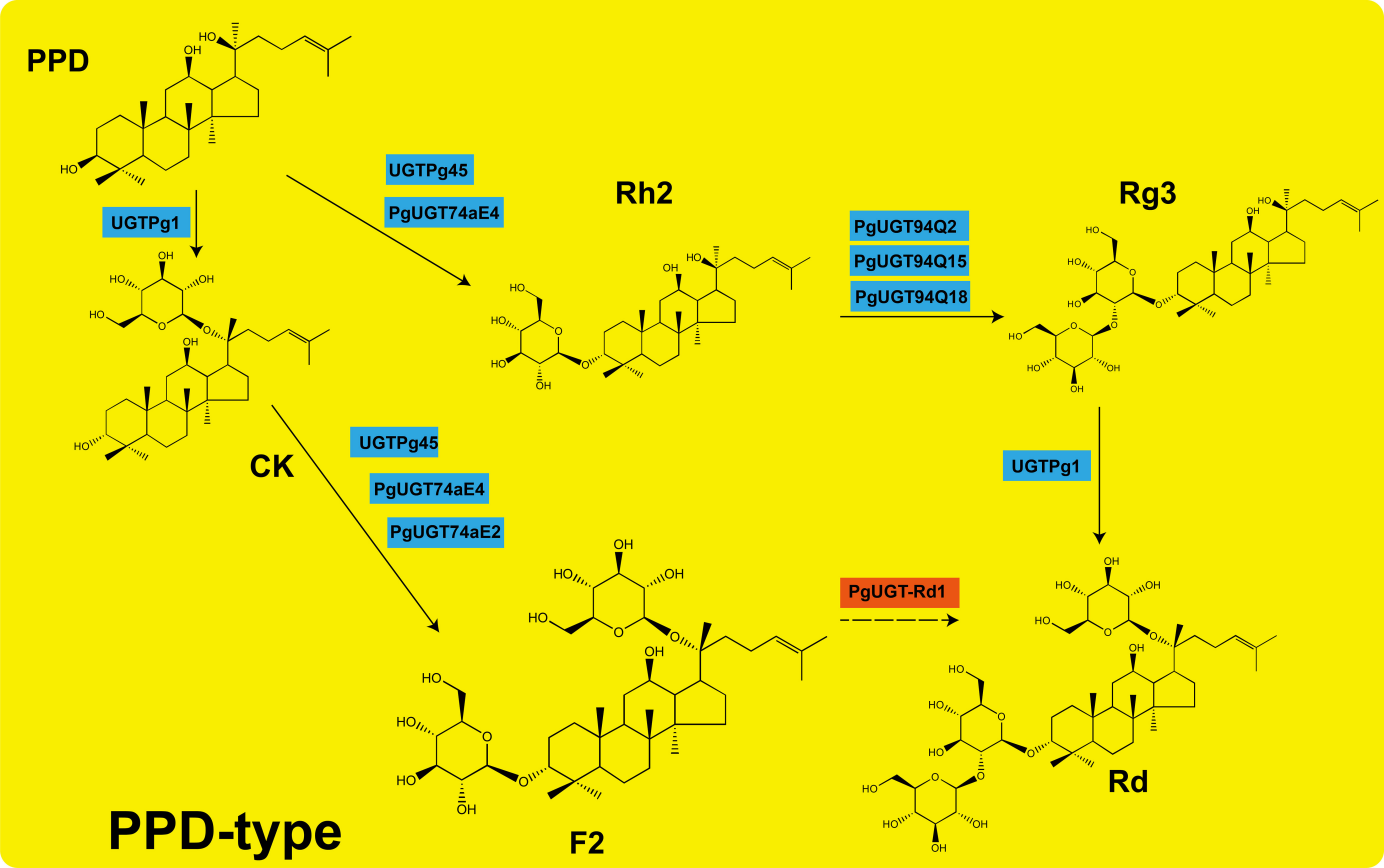
Proposed biosynthetic pathway of ginsenoside Rd and the role of PgUGT-Rdl.

This schematic outlines the PPD-type ginsenoside branch in P. ginseng. Protopanaxadiol (PPD) and protopanaxatriol (PPT) are the two primary dammarane aglycones derived from dammarenediol-II. Ginsenoside CK (Compound K) is a PPD-type intermediate with a glucose at the C20 position, whereas ginsenoside Rh2 has a glucose at the C3 position. Prior studies identified PgUGT74AE2 as an enzyme that glucosylates PPD at C3 (or CK at C3) to form Rh2 and F2, respectively. The novel gene *PgUGT-Rd1* (designated here as *PgUGT-Rd1*) encodes a UDP-glycosyltransferase that we propose catalyzes the addition of a second glucose to ginsenoside F2, converting it into ginsenoside Rd (which has two glucose units at C20 and one at C3). By the same reaction, *PgUGT-Rd1* may also add a glucose to Rh2 (which has one glucose at C3) to produce ginsenoside Rg3 (two glucose units at C3). In the diagram, the step facilitated by *PgUGT-Rd1* is highlighted with a bold arrow. Enzyme names in italics indicate characterized ginseng glycosyltransferases: PgUGT74AE2 (C3 glycosylation) and PgUGT94Q2 (another ginseng UGT known to form Rg3 and Rd *in vitro*). The discovery of *PgUGT-Rd1* enriches this pathway by identifying a previously uncharacterized UGT responsible for the terminal glycosylation step leading to Rd. Further downstream, additional glycosylations (dashed arrows) convert Rg3 and Rd into higher oligoglycosides such as ginsenoside Rb1 (not shown for simplicity). This pathway diagram underscores the central position of *PgUGT-Rd1* in specifically directing flux toward Rd biosynthesis in ginseng. (**Figure 10**).

Mechanistically, an intriguing question is how *PgUGT-Rd1*, being an enzyme, exerts a regulatory influence on other biosynthetic genes. We propose several, non-mutually-exclusive hypotheses: (1) Metabolic feedback regulation: *PgUGT-Rd1* catalyzes the formation of ginsenoside Rd (or a Rd precursor), and the accumulation of this product (or depletion of its substrate) could feed back to regulate the pathway. In plant specialized metabolism, it is common for the end products or key intermediates to act as feedback signals, modulating the expression of upstream genes. In this case, a highly active *PgUGT-Rd1* allele might drive Rd accumulation to levels that trigger a feedback response, up-regulating or down-regulating certain genes to maintain metabolic balance. Conversely, a weak *PgUGT-Rd1* could lead to buildup of upstream intermediates, which might signal the plant to adjust expression of those pathway enzymes. (2) Signaling molecule production: It is also possible that the glycosylation catalyzed by *PgUGT-Rd1* produces not just Rd for storage, but also bioactive glycosides that function as signal molecules. Some glycosylated triterpenes or their derivatives might act in hormone signaling pathways or as messengers in stress responses. If *PgUGT-Rd1*’s activity influences the pool of such signaling compounds, it could indirectly affect transcriptional networks – for example, by modulating jasmonate signaling or other hormone circuits that broadly induce or repress biosynthetic genes. This could explain why *PgUGT-Rd1* expression is tightly coupled with other genes under MeJA induction regimes. (3) Transcriptional cross-talk or co-regulation: Another possibility is that *PgUGT-Rd1* and the other Rd-pathway genes are co-regulated by one or more upstream transcription factors. In this scenario, *PgUGT-Rd1* might not physically regulate those genes, but its expression is an indicator of a broader transcriptional program activated by certain transcription factors (for instance, AP2/ERF, bHLH, or MYB family regulators known to control secondary metabolism in plants). If a transcription factor activates *PgUGT-Rd1* alongside other pathway enzymes, *PgUGT-Rd1* becomes part of a positive feedback loop: its enzymatic product (Rd) might stabilize the regulatory network or further stimulate the transcription factor’s activity (a form of feed-forward regulation). (4) Protein–protein interactions or metabolon formation: Although speculative, *PgUGT-Rd1* might form part of a multi-enzyme complex (metabolon) or interact with regulatory proteins, helping to channel substrates efficiently through the Rd branch or recruiting transcriptional machinery to certain genes. Some metabolic enzymes have non-catalytic “moonlighting” roles in scaffolding or signaling, and *PgUGT-Rd1* could potentially have such dual functionality. While our data cannot distinguish among these mechanisms, the strong co-expression and the clear impact of perturbing *PgUGT-Rd1* (via MeJA induction or RNAi) on the entire pathway support the idea that *PgUGT-Rd1* is intricately linked to pathway regulation. Targeted experiments (discussed below) will be required to unravel the exact mechanism of this regulatory influence.

Furthermore, our network framework offers insight into metabolic branch competition and environmental modulation of ginsenoside biosynthesis. The Rd biosynthetic genes do not operate in isolation; they interface with other ginsenoside branches (e.g., the protopanaxatriol branch) and are subject to regulation by external cues. By positioning *PgUGT-Rd1* within a co-expression network, we gained a clearer view of how the PPD-type (Rd) branch may compete with or complement other branches for shared precursors, and how it might be coordinately regulated. The tight association of *PgUGT-Rd1* with both structural genes and putative regulators suggests that environmental factors like hormones and abiotic stresses could influence Rd production by acting on this network hub. Indeed, transcriptional regulatory modules responding to stimuli (such as jasmonates, which mimic stress signals) likely converge on nodes like *PgUGT-Rd1* to modulate flux through the pathway. This improved understanding provides a theoretical foundation for strategies to stabilize or enhance the production of pharmacologically active ginsenosides under varying growth conditions. For instance, with knowledge of the network, one could aim to breed or engineer ginseng lines that maintain strong *PgUGT-Rd1* network connectivity even under stress or in poor environments, thereby ensuring more consistent Rd yields. In essence, our constructed Rd biosynthetic network not only advances current knowledge of secondary metabolism in ginseng, but also serves as a paradigm for linking genotype, gene network architecture, and metabolic phenotype. It illustrates how a single key gene, embedded in its network context, can be leveraged to improve metabolite production and consistency across diverse conditions.

Despite the strengths of our study, there are several limitations that warrant discussion, which also point to directions for future research. **First**, while our correlation analyses and functional tests (MeJA induction and RNAi silencing) implicate *PgUGT-Rd1* as a central regulator, we did not fully elucidate the molecular mechanism by which *PgUGT-Rd1* influences other genes or the overall flux. We inferred regulation largely from co-expression and indirect evidence; thus, it remains to be determined whether *PgUGT-Rd1* controls other pathway genes through metabolic feedback, signaling, or shared transcriptional regulators (as hypothesized above). Future experiments should address this gap. For example, a combined MeJA treatment and *PgUGT-Rd1* RNAi experiment could be informative: if exogenous MeJA fails to restore Rd levels or upstream gene expression in *PgUGT-Rd1*-silenced plants, this would suggest *PgUGT-Rd1* acts downstream of (or is essential for) the jasmonate-responsive pathway activation. Conversely, if MeJA can partially rescue Rd production even when *PgUGT-Rd1* is knocked down, it might indicate parallel or compensatory pathways. Additionally, transcriptomic analyses of plants or cells with *PgUGT-Rd1* knocked out or overexpressed would help identify differentially expressed genes and pathways, shedding light on what downstream effects *PgUGT-Rd1* exerts. Such RNA-sequencing data, especially when coupled with metabolomic profiling, could detect signatures of feedback inhibition or activation (for instance, accumulation of upstream intermediates might repress or induce certain genes, which would appear in the transcriptome). Protein interaction assays represent another important avenue: Co-immunoprecipitation or yeast two-hybrid screens could test whether *PgUGT-Rd1* interacts with any candidate transcription factors or other enzymes, exploring the possibility of a physical complex or signaling interaction. If *PgUGT-Rd1* were found to associate with a known regulator of secondary metabolism, that would provide a direct mechanistic link between its enzymatic function and transcriptional control of the pathway. We also acknowledge that our study focused on transcriptional networks and enzyme function, but did not examine post-translational modifications or enzyme kinetics in detail—factors which could significantly affect *PgUGT-Rd1* activity and its regulatory impact. Investigating whether *PgUGT-Rd1* is subject to phosphorylation or other modifications (perhaps by kinases activated under stress) could reveal additional layers of regulation. Second, our co-expression network analysis, while comprehensive, provides a static snapshot based on the conditions and genotypes sampled. Ginseng’s response to environmental variables (soil, climate, biotic stress) over time remains an open question. Future studies could employ time-course experiments or multi-environment trials to see how the *PgUGT-Rd1* network behaves under different conditions, thereby validating the network’s robustness and identifying any condition-specific co-regulators. Third, the translational aspect—leveraging *PgUGT-Rd1* for metabolic engineering—still requires practical demonstration. Building a yeast or microbial production system for Rd, for instance, will require not only transferring *PgUGT-Rd1* and other pathway genes but also tuning their expression and possibly modifying the host’s metabolism. Our study lays the groundwork by identifying the key players, but iterative engineering and optimization will be needed to turn this knowledge into a viable production platform. Finally, while we demonstrated the utility of *PgUGT-Rd1* as a breeding marker, actual breeding programs will need to consider the gene’s effects in diverse genetic backgrounds and over multiple generations. It will be important to confirm that the favorable alleles of *PgUGT-Rd1* consistently lead to higher Rd content without undesirable pleiotropic effects on plant growth or other traits. Marker-assisted selection or genomic selection incorporating *PgUGT-Rd1* allelic status should be tested in practice to quantify the improvement in breeding efficiency for ginsenoside traits.

In conclusion, our integrative dissection of *PgUGT-Rd1* and its co-expression network has substantially advanced the understanding of Rd-specific biosynthesis in ginseng. We have identified *PgUGT-Rd1* as a central regulatory node whose expression and allelic variation govern Rd accumulation, and we mapped a network of interacting genes that collectively drive this valuable metabolic pathway. These findings fill a critical gap in ginsenoside research and provide actionable targets for improving ginseng through molecular breeding and metabolic engineering. By illuminating the genetic and regulatory architecture of ginsenoside Rd biosynthesis, this work offers a new paradigm for connecting natural variation to specialized metabolism in medicinal plants, and paves the way for the efficient and sustainable production of specific ginsenosides in planta or in engineered biological systems. The insights gained here not only deepen scientific knowledge of plant secondary metabolism but also hold practical promise for enhancing the pharmacological value of ginseng and related species in the years to come.

## 5 Result

Using an integrated multi-omics strategy, this study systematically identified key genes and regulatory factors closely associated with ginsenoside Rd content in ginseng and established a preliminary regulatory network for Rd biosynthesis. We discovered and functionally validated *PgUGT-Rd1* as a core gene whose expression level significantly affects Rd accumulation and whose genetic variation exhibits clear phenotypic effects, indicating that it plays an important rate-limiting regulatory role within the PPD-type ginsenoside branch pathway. In addition, through GWAS and co-expression analyses, we further confirmed the functional status of key enzyme genes such as *CYP716A53v2* and *UGT71A27*, thereby demonstrating the strong applicability and transferability of a multi-omics integration strategy centered on individual ginsenoside monomers.

Overall, these findings provide new experimental evidence for the genetic and molecular mechanisms underlying Rd biosynthesis, and they offer critical targets and practical tools for molecular breeding and metabolic engineering of ginseng. They also lay a theoretical foundation for the efficient production of specific ginsenoside monomers in heterologous systems.

## Data Availability

The datasets presented in this study can be found in online repositories. The names of the repository/repositories and accession number(s) can be found in the article/**Supplementary Material**.
