# Supplementary figures and images for: Genome-wide and co-expression network dissection of PgUGT-Rd1 as a central regulator of ginsenoside Rd biosynthesis in ginseng
Source: Front Plant Sci. 2026 Mar 2;17:1751774. doi: 10.3389/fpls.2026.1751774 (PMC12989552; doi:10.3389/fpls.2026.1751774)

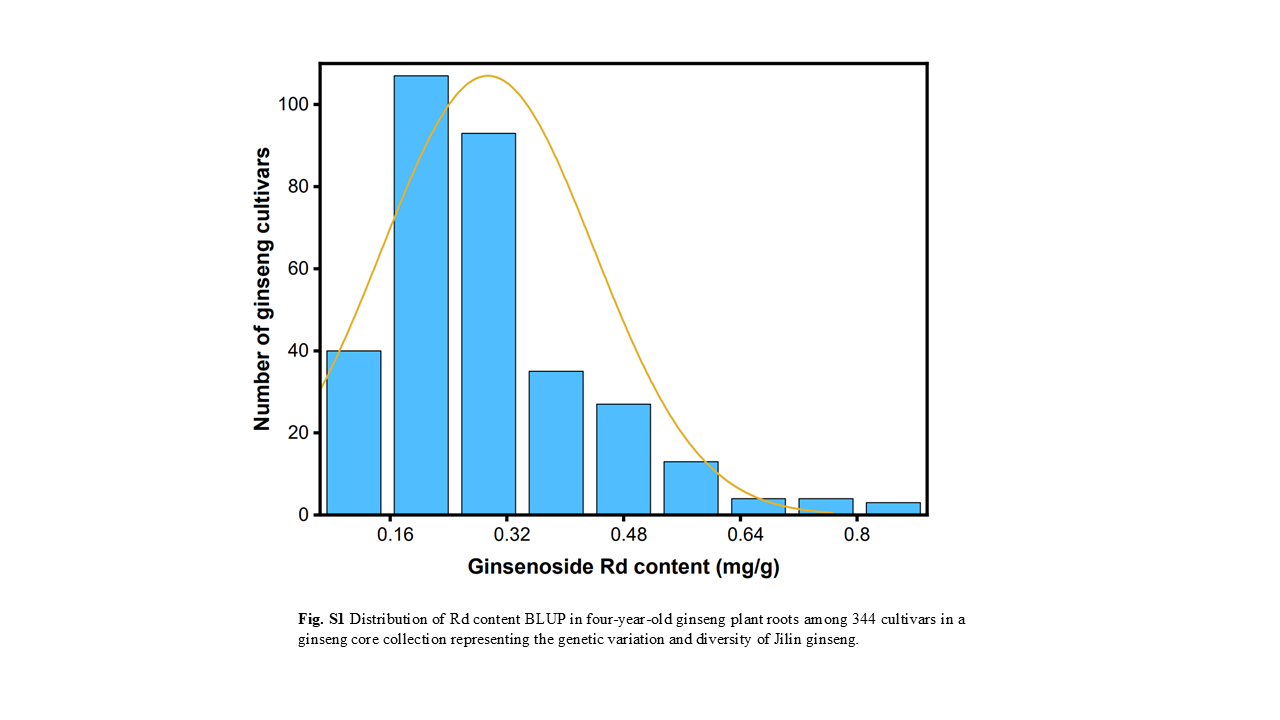

Supplement: Supplementary file 1 [file Image1.tif]

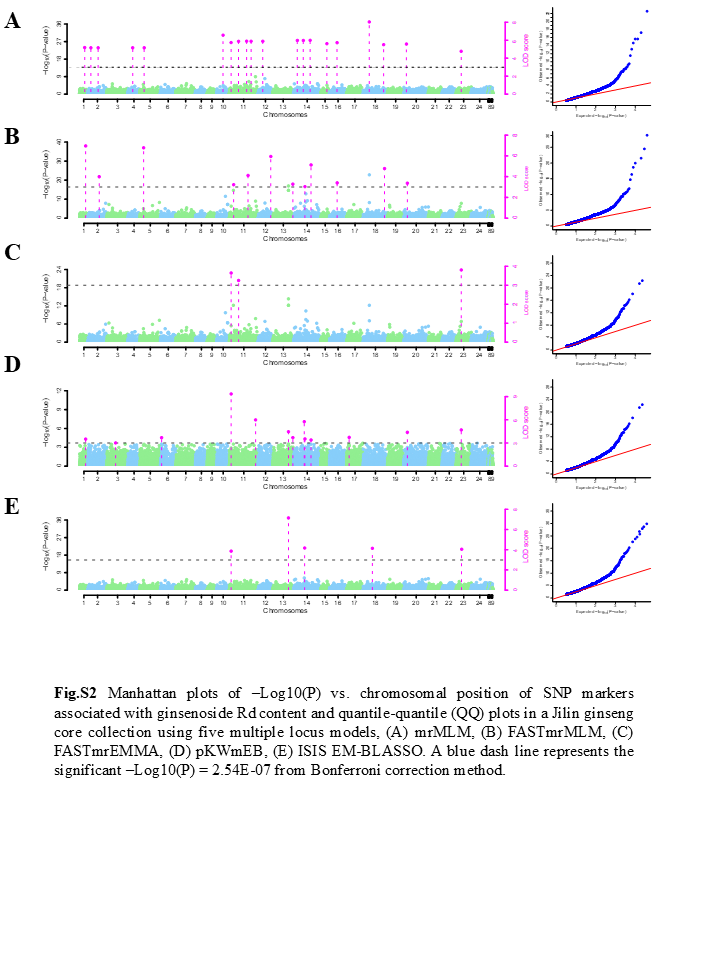

Supplement: Supplementary file 2 [file Image2.tif]

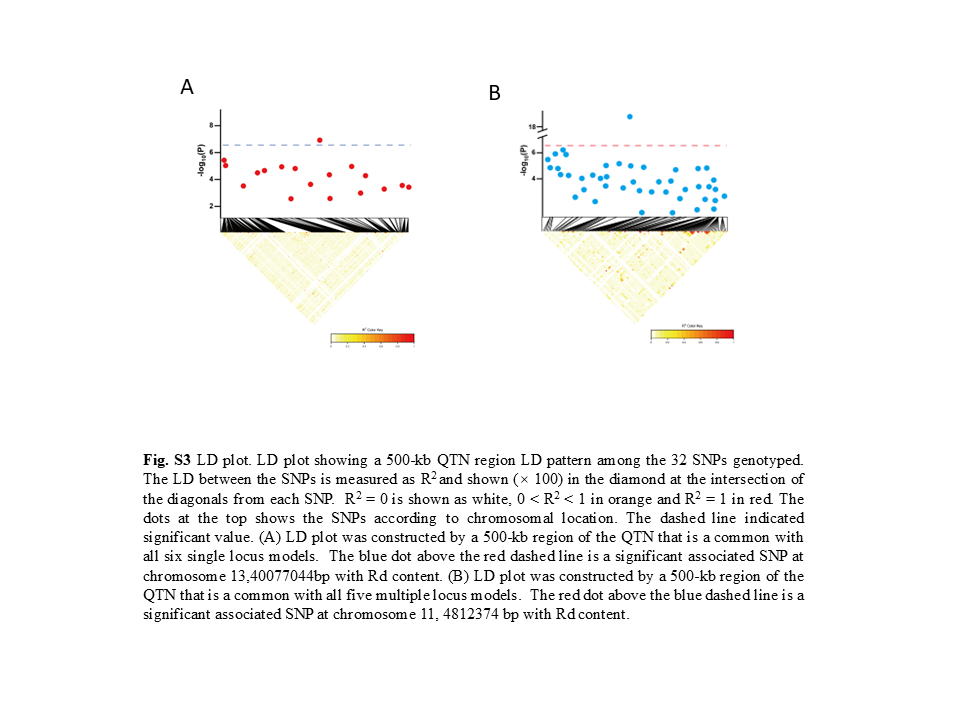

Supplement: Supplementary file 3 [file Image3.tif]

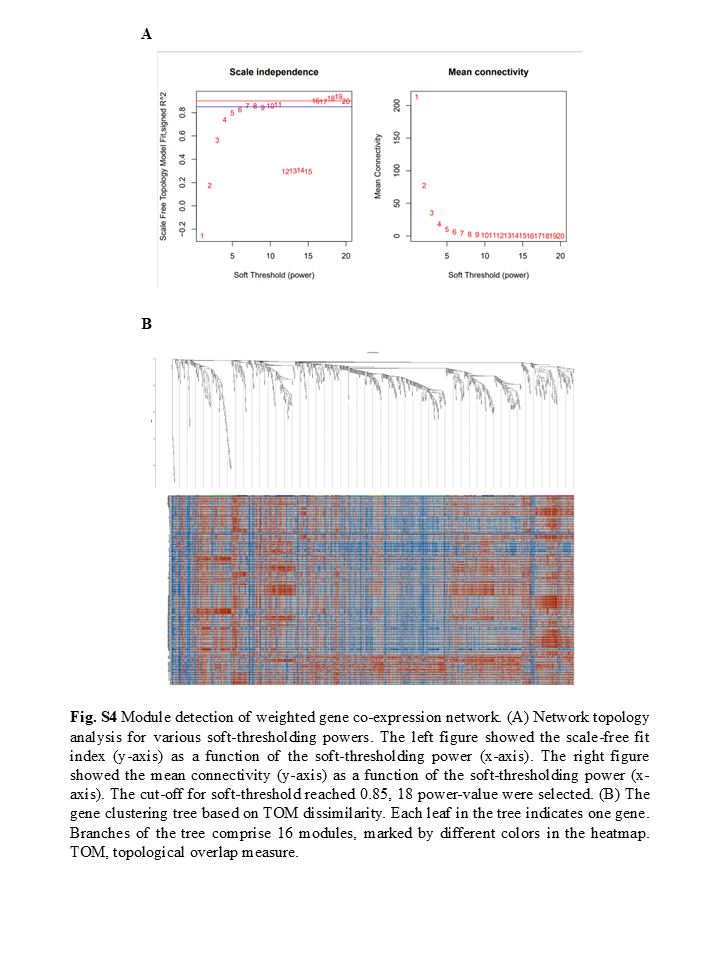

Supplement: Supplementary file 4 [file Image4.tif]

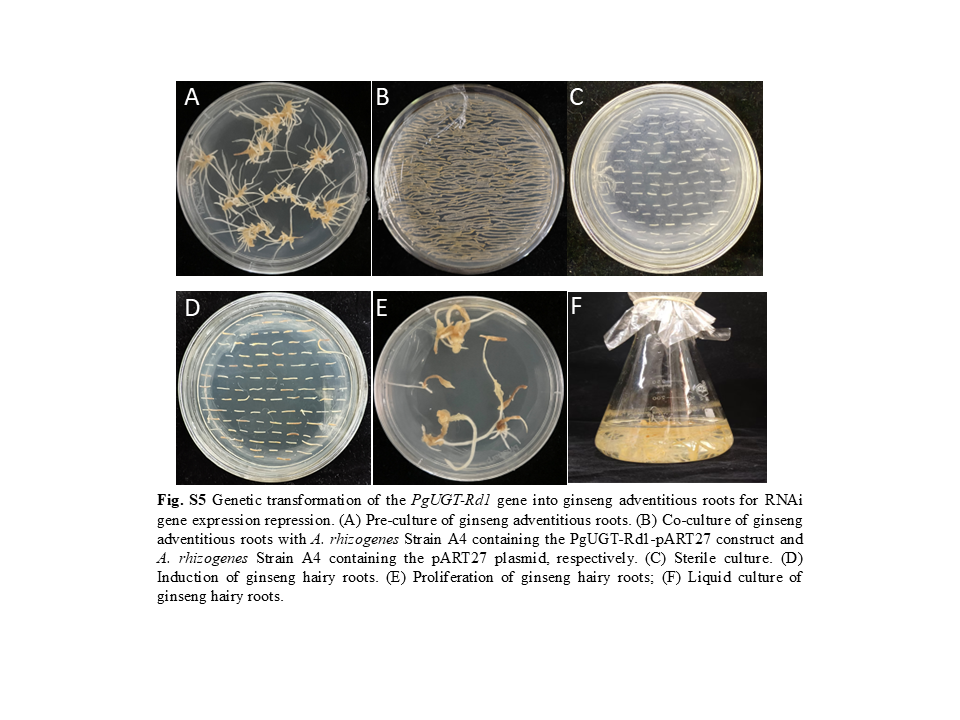

Supplement: Supplementary file 5 [file Image5.tif]

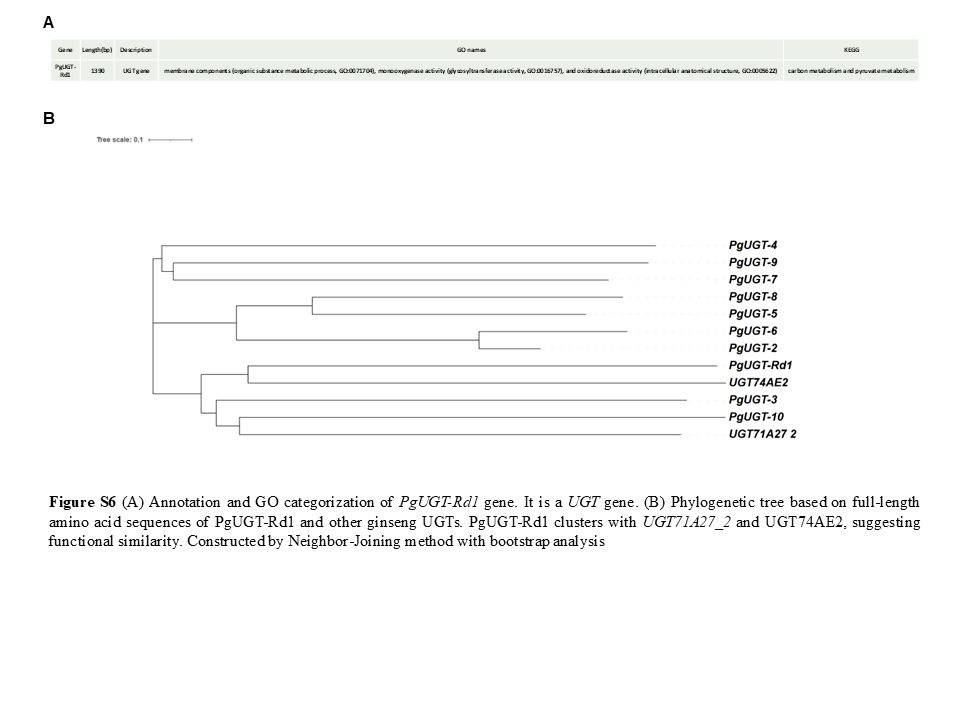

Supplement: Supplementary file 6 [file Image6.tif]

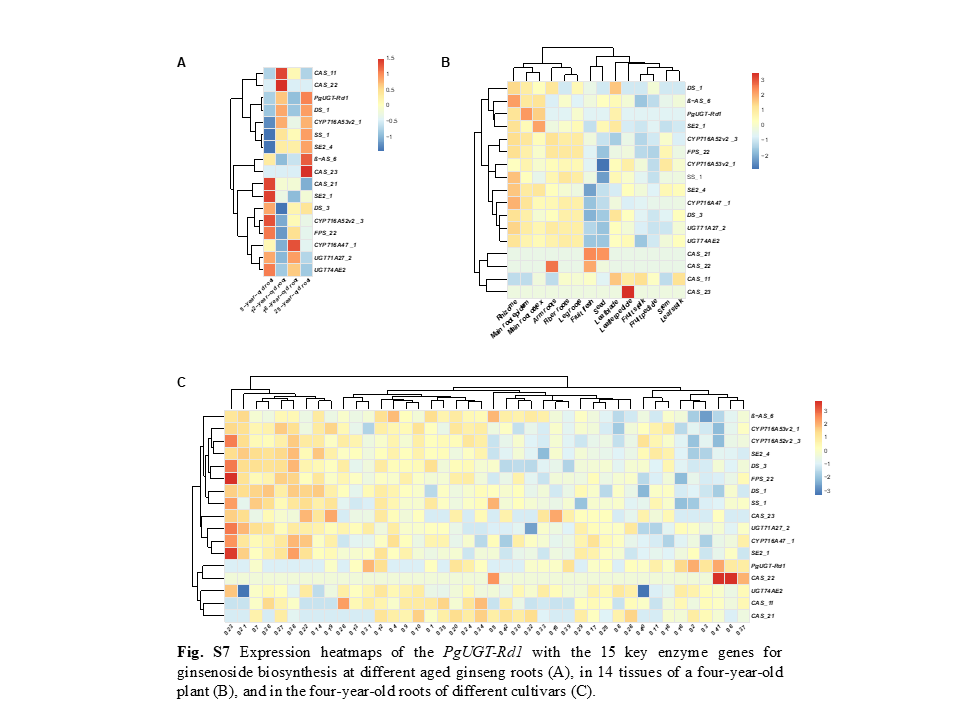

Supplement: Supplementary file 7 [file Image7.tif]
